# Inferring Neuronal Dynamics from Calcium Imaging Data Using Biophysical Models and Bayesian Inference

**DOI:** 10.1371/journal.pcbi.1004736

**Published:** 2016-02-19

**Authors:** Vahid Rahmati, Knut Kirmse, Dimitrije Marković, Knut Holthoff, Stefan J. Kiebel

**Affiliations:** 1 Department of Psychology, Technische Universität Dresden, Dresden, Germany; 2 Biomagnetic Centre, Hans-Berger Department of Neurology, University Hospital Jena, Jena, Germany; 3 Hans-Berger Department of Neurology, University Hospital Jena, Jena, Germany; 4 Max Planck Institute for Human Cognitive and Brain Sciences, Leipzig, Germany; Hamburg University, GERMANY

## Abstract

Calcium imaging has been used as a promising technique to monitor the dynamic activity of neuronal populations. However, the calcium trace is temporally smeared which restricts the extraction of quantities of interest such as spike trains of individual neurons. To address this issue, spike reconstruction algorithms have been introduced. One limitation of such reconstructions is that the underlying models are not informed about the biophysics of spike and burst generations. Such existing prior knowledge might be useful for constraining the possible solutions of spikes. Here we describe, in a novel Bayesian approach, how principled knowledge about neuronal dynamics can be employed to infer biophysical variables and parameters from fluorescence traces. By using both synthetic and *in vitro* recorded fluorescence traces, we demonstrate that the new approach is able to reconstruct different repetitive spiking and/or bursting patterns with accurate single spike resolution. Furthermore, we show that the high inference precision of the new approach is preserved even if the fluorescence trace is rather noisy or if the fluorescence transients show slow rise kinetics lasting several hundred milliseconds, and inhomogeneous rise and decay times. In addition, we discuss the use of the new approach for inferring parameter changes, e.g. due to a pharmacological intervention, as well as for inferring complex characteristics of immature neuronal circuits.

## Author Summary

Calcium imaging of single neurons enables the indirect observation of neuronal dynamics, for example action potential firing. In contrast to the precise timing of spike trains, the calcium trace is temporally rather smeared and measured as a fluorescence trace. Consequently, several methods have been proposed to reconstruct spikes from calcium imaging data. However, a common feature of these methods is that they are not based on the biophysics of how neurons fire spikes and bursts. We propose to introduce well-established biophysical models to create a direct link between neuronal dynamics, e.g. the membrane potential, and fluorescence traces. Using both synthetic and experimental data, we show that this approach not only provides a robust and accurate spike reconstruction but also a reliable inference about the biophysically relevant parameters and variables. This enables novel ways of analyzing calcium imaging experiments in terms of the underlying biophysical quantities.

## Introduction

Calcium imaging has been used to record neuronal activity indirectly [1]. Individual neurons or neuronal populations are labeled by applying fluorescent calcium indicators so that the intracellular calcium transients become measurable by optical means. The technique has a temporal resolution up to the millisecond scale and allows measurements at multiple structural levels, from cellular sub-compartments [2,3] and individual neurons [4,5] to rather large neuronal ensembles [6–8]. Moreover, calcium imaging can be applied to both *in vivo* [6,9,10] and *in vitro* brain preparations [11,12].

While calcium imaging techniques have a wide range of applications, the reconstruction of the quantities of interest such as spike (or action potential) trains and intracellular free calcium concentration, [Ca^2+^], kinetics from fluorescence, *F*_*t*_, traces is restricted mostly due to the intrinsic low temporal resolution of calcium traces, relative to the fast time scale of spikes. Several algorithms for the reconstruction of spikes from calcium imaging data have been proposed. These include template matching [7,9,13,14], first-derivative methods including deconvolution [15–17], reverse correlation [18] and a sequential Monte Carlo method [19].

One common feature of these previously described reconstruction methods is that they are not based on biophysical models for how spikes are generated. Such biophysical models have been developed to model different biophysical characteristics and firing patterns observed in electrophysiological data [20,21]. Here, we specify a generative forward model by explicitly linking the membrane potential (*V*), rather than only spikes, to fluorescence traces through [Ca^2+^] kinetics. We pursue the idea that reconstruction of spikes and, potentially, inferring the underlying neuronal dynamics, can be performed by inverting this generative forward model. The inversion of such biophysically plausible models has been found useful for magneto- and electroencephalography and can be performed using Bayesian inference techniques [22,23]. The expected advantages for the analysis of calcium imaging data, relative to existing approaches, are an increased robustness to noise and artefacts, incorporation of biophysically sensible prior information, and the biophysical interpretation of inferred variables and identified parameters. In addition, the Bayesian inference approach captures the uncertainty about the inferred quantities of interest, thereby allowing one to assess which dynamics and parameters can be inferred from the fluorescence traces, and which ones cannot.

As an illustration and proof-of-concept of the proposed modelling approach, we use both synthetic and *in vitro* calcium imaging data. We show for both data sets that spikes can be reconstructed accurately, even under low signal to noise (SNR) conditions, through inferring the neuronal dynamics such as membrane potential, [Ca^2+^] kinetics, membrane refractoriness and voltage-gated ionic currents. One important, potential application of the approach may be its use to reconstruct spikes from imaging data at high sampling rates but with rather low SNRs. We will show that the biophysical model reliably infers the within-burst or single spikes and also quantitatively captures and infers several experimental phenomena, e.g. fluorescence transients with both typical (i.e. fast) [13,24–26] or slow rise kinetics [27], and the somatic bursts of hippocampal pyramidal neurons [28–30]. We will further demonstrate the usefulness of the approach for experimental setups like a pharmacological intervention where predictions about the change in specific biophysical parameters can be tested directly. To illustrate the method on real data, we use our recorded data from immature neurons of neonatal mice which impose a challenging spike reconstruction task, due to the slow rise kinetics of their spike-evoked fluorescence transients. In addition, we apply the method to a publicly available *in vitro* data set with fast rising fluorescence transients. To reduce the computational complexity of the method we also introduce and use two new, differentiable integrate-and-fire models for repetitive spiking and bursting firing patterns.

## Methods

### Overview

**Fig 1:**
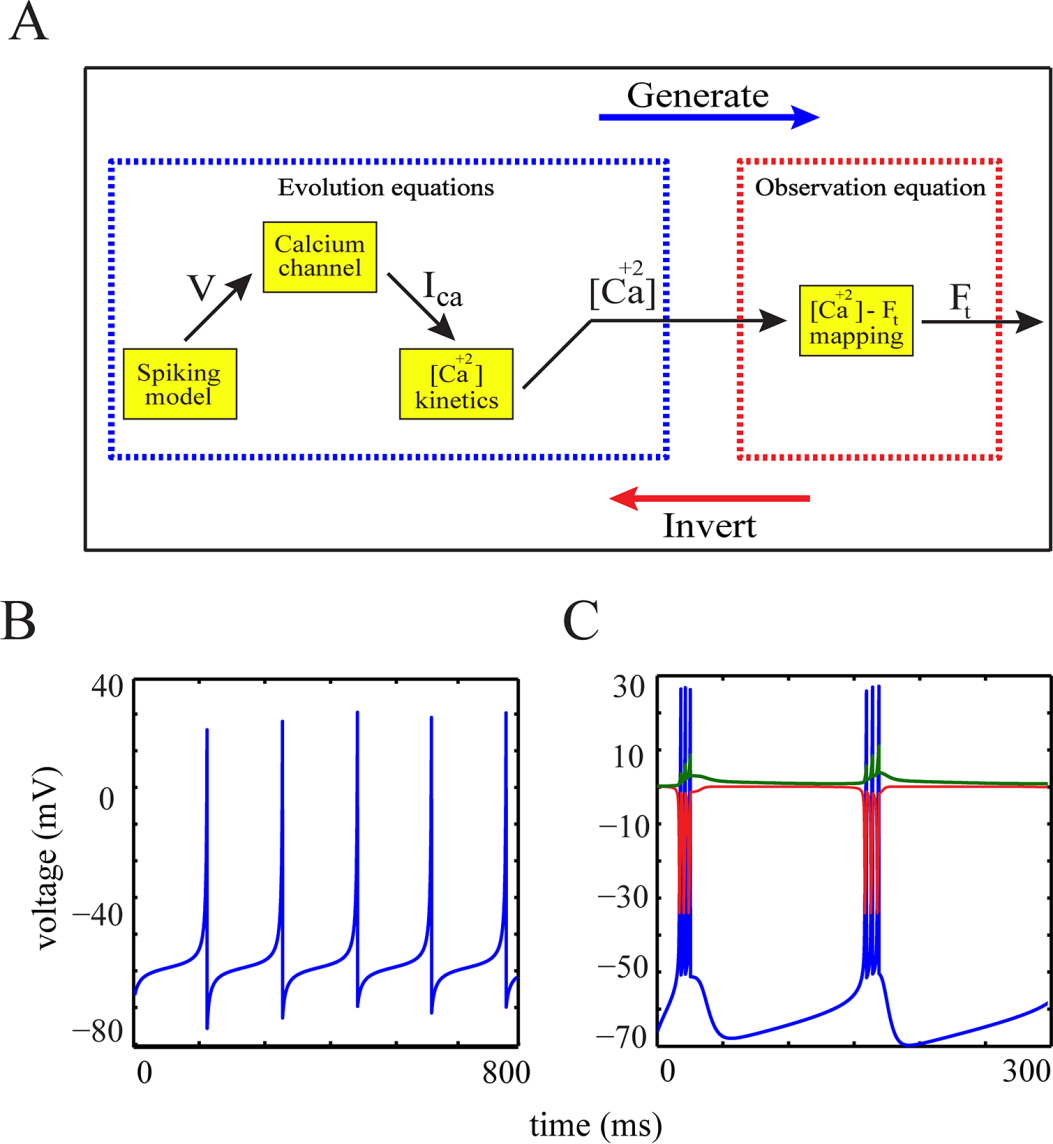
The generative model and non-hybrid quadratic integrate-and-fire (family of QGIF) models. (**A**) Graph representing the generative model and its inversion, which are comprised of evolution (i.e., a neuron model) and observation equations. The illustrated hierarchy in the graph displays how neuronal dynamics relate to fluorescence traces. (**B** and **C**) Sample voltage traces of (**B**) QGIF and (**C**) bursting-QGIF models. The traces show the rhythmic activity of these models in response to sustained depolarizations. Note that the family of QGIF models does not require any reset condition for spike/burst generation. The persistent Na^+^ and M-type K^+^ currents (red and green lines, respectively) of bursting-QGIF model are in units of [*μA* / *cm*^2^]. Parameters for the simulations: (**B**) *I*_*app*_ = 0.2 *μA* / *cm*^2^, (**C**) ${\bar{I}}_{rep}=80\;\mu A\;mS/\mu F$, *I*_*app*_ = 1 *μA* / *cm*^2^. See Table 1 for the rest of parameter values.

Our approach for model-based analysis of calcium imaging data is based on linking the neuronal membrane potential, to the fluorescence traces by using the kinetics of [Ca^2+^] as an intermediate variable. This requires three components: (i) a model for the membrane dynamics and how spikes are generated, (ii) a model for the [Ca^2+^] kinetics, where the entry via Ca^2+^ influx is regulated by a nonlinear function representing the activation-state of high-voltage-activated (HVA) calcium channels, and (iii) an observation function which provides a noisy nonlinear mapping from the generated [Ca^2+^] time series to the fluorescence trace. These three model components form a so-called generative model which explains how data are generated starting from the membrane potential. Given a fluorescence trace, we ‘invert’ this model by making inference on the neuron’s underlying hidden states (i.e. dynamics) and/or parameters, see Fig 1A.

**Table 1:** Parameter values.

Models	Parameters	Units	References
**QGIF family**	*V*_*th*_ = −59.9	*mV*	[48]
	*I*_*th*_ = 0.16	*μA* / *cm*^2^	[48]
	*g*_*L*_ = 0.1	*mS* / *cm*^2^	[48]
	*C* = 1	*μF* / *cm*^2^	[48]
	*V*_*peak*_ = 30	*mV*	[29]
	*σ*_*peak*_ = 1	*mV*	
	*a*_*rep*_ = 10^12^	*mV*	
	*b*_*rep*_ = 10^4^		
	*C*_*rep*_ = 0.1		
**QGIF**	${\bar{I}}_{rep}=120$	*μA mS* / *μF*	
	Δ_*th*_ = 3.48	*mV*	[48]
	*dt* = 0.2	*ms*	[21]
	*k* = 3 **[INV]**		
	ϒ_*F*_ = 3 **[INV]**		
	ϒ_*V*_ = 0.005 **[INV]**		
	ϒ_*Ca*_ = 10 **[INV]**		
	$\tau_{Ca}^{real}=2000$ **[SIM]**	*ms*	
	$\tau_{Ca}^{real}=7500$ **[INV]**	*ms*	
**bursting-QGIF**	${\bar{I}}_{rep}=87$ **[SIM]**	*μA mS* / *μF*	
	${\bar{I}}_{rep}=80$ **[INV]**		
	*E*_*K*_ = −90	*mV*	[57]
	*E*_*Na*_ = 55	*mV*	[57]
	*g*_*M*_ = 1 **[U]**	*mS* / *cm*^2^	[57]
	*g*_*NaP*_ = 0.41 **[SIM] [U]**	*mS* / *cm*^2^	[57]
	Δ_*th*_ = 3.48 **[SIM] [U]**	*mV*	[48]
	*O* = 0		
	*dt* = 0.05	*ms*	
	*k* = 8 **[INV]**		
	ϒ_*F*_ = 6 **[INV]**		
	ϒ_*V*_ = 0.005 **[INV]**		
	ϒ_*z*_ = 20 **[INV]**		
	ϒ_*Ca*_ = 10 **[INV]**		
	$\tau_{Ca}^{real}\approx 800$ **[SIM]**	*ms*	
	$\tau_{Ca}^{real}=850$ **[INV]**	*ms*	
**FHN**	*ψ*_1_ = 0.7		[21]
	*ψ*_2_ = 0.8		[21]
	*λ* = 0.08		[21]
	*dt* = 0.2	*ms*	[21]
	*k* = 3 **[INV]**		
	ϒ_*F*_ = 3 **[INV]**		
	ϒ_*V*_ = 2 **[INV]**		
	ϒ_*W*_ = 30 **[INV]**		
	ϒ_*Ca*_ = 10 **[INV]**		
	$\tau_{Ca}^{real}=2000$ **[SIM]**	*ms*	
	$\tau_{Ca}^{real}=7500$ **[INV]**	*ms*	
**Calcium channel**	*g*_*Ca*_ = 5	*mS* / *cm*^2^	[29]
	*κ*_*Ca*_ = 0.002 **[SIM] [U]**		[64]
	[Ca^2+^]_base_ = 0 **[SIM] [U]**		
	*E*_*Ca*_ = 120	*mV*	[64]
	*ρ* = 5	*mV*	[64]
	*V*_1/2_ = −25 **[SIM]**	*mV*	[64]
	*V*_1/2_ = −45 **[INV]**	*mV*	[67]
**Ca**^**2+**^ **-fluorescence mapping**	*K*_*d*_ = 200 **(for OGB-1)**	*nM*	[4]
	*κ*_*F*_ = 10 **[U] (for QGIF)**		
	*κ*_*F*_ = 5 **[U] (for bursting-QGIF & FHN)**		
	*d*_*F*_ = 0 **[U]**		
**Precision-parameters**	*σ* = inf ≈ 10^12^ **[SIM] [U]**		
	*α* = inf ≈ 10^12^ **[SIM] [U]**		

Parameter values used in the generative and inverse models. This table lists the default values of modeling parameters. The abbreviations **SIM** and **INV** in front of the parameter values (second column) indicate that these values were used in the simulations and inversions, respectively. The parameters designated by **U** were considered as free parameters in the inversions and thus need to be inferred from the data; the prior distributions for these parameters are listed in Table 2. Moreover, for simulating data the initial conditions of all neuronal dynamics were set to their steady-states in the absence of input to neuron model. The rest of parameters were kept with the same values in both simulations and inversions. All parameter values are consistent across the figures, unless stated otherwise.

**Table 2:** Prior densities.

Models	Parameters	Prior densities
**All**	$\tau_{Ca}=\tau_{Ca}^{0}\exp\left(\chi_{\tau_{Ca}}\right)$	$\chi_{\tau_{Ca}}∼N(0,5)$
	$\kappa_{Ca}=0.002\exp\left(\chi_{\kappa_{Ca}}\right)$	$\chi_{\kappa_{Ca}}∼N(0,5)$
	[Ca^2+^]_base_	[Ca^2+^]_base_ ∼ *N*(0,100)
	[Ca^2+^](0)	$\left[{\text{Ca}}^{2+}](0)∼N({\left[{\text{Ca}}^{2+}\right]}_{}^{ss}{|}_{V_{1/2}=-45},25\right)$
	$\kappa_{F}=5\exp(\chi_{\kappa_{F}})$	$\chi_{\kappa_{F}}∼N(0,1)$
	*d*_*F*_	*d*_*F*_ ∼ *N*(0.5min(*F*_*t*_),0.25)
	*α*	*α* ∼ *Ga*(1,1)
	*σ*	*σ* ∼ *Ga*(1,1)
**QGIF**	*V*(0)	*V*(0) ∼ *N*(*V*^*ss*^,100)
**Bursting-QGIF**	$\Delta_{th}=3.48\exp\left(\chi_{\Delta_{th}}\right)$	$\chi_{\Delta_{th}}∼N(0,1)$
	$g_{NaP}=0.41\exp\left(\chi_{g_{NaP}}\right)$	$\chi_{g_{NaP}}∼N(0,1)$
	$g_{M}=\exp\left(\chi_{g_{M}}\right)$	$\chi_{g_{M}}∼N(0,1)$
	*V*(0)	*V*(0) ∼ *N*(*V*^*ss*^,100)
	*z*(0)	*z*(0) ∼ *N*(*z*^*ss*^,1)
**FHN**	*V*(0)	*V*(0) ∼ *N*(*V*^*ss*^,4)
	*W*(0)	*W*(0) ∼ *N*(*W*^*ss*^,1)

Prior densities of the free (i.e. unknown) parameters. *N*(mean,variance) indicates normal distribution, and *Ga*(shape, rate) indicates a Gamma distribution. The superscript *ss* denotes the steady-state value of the corresponding variable in the absence of input to neuron model. The proper values for $\tau_{Ca}^{0}$ (in terms of $\tau_{Ca}^{real}$) can be found in Table 1.

For inference, we use a recently developed approximate (variational) Bayesian inference approach [31] for stochastic, nonlinear state-space models, i.e. discretized stochastic nonlinear dynamic systems. In the following, we refer to this variational Bayesian approach as VB-Laplace. This approach performs efficient and robust parameter estimation even if both the evolution and observation equations (as with the proposed calcium imaging model) are nonlinear. As the framework enables analytical update equations, the method is generic and efficient in terms of computation time. Critically, the approach also enables the estimation of parameters (e.g., calcium decay time-constant) and precision-parameters, e.g. noise level on fluorescence trace. As we will show below, this is important for the application to calcium imaging data. As with any Bayesian approach, the method relies on the specification of the prior distributions of the parameters. This is useful because the generative model is biophysical and plausible prior knowledge can be derived directly from previous modelling and experimental studies.

The generative model is defined in terms of its state (i.e. evolution) and observation (i.e. likelihood) equations:

$$\text{Generative model}:\{\begin{array}{l} x_{t+1}=f\left(x_{t},\theta ,u_{t}\right)+\eta_{t}\\ y_{t}=g\left(x_{t},\varphi ,u_{t}\right)+\varepsilon_{t} \end{array}$$ (1)

where *x* denote the states of neuronal dynamics such as *V* and [Ca^2+^], *y*_*t*_ refers to the fluorescence responses, *θ* and *φ* are quantities that parameterize the state evolution function *f* and observation function *g*, respectively, and *u*_*t*_ is the synaptic (or applied) input time series to the membrane. In this generic formalism, *η*_*t*_ denotes state-noise with precision (i.e., inverse variance) *α* and *ε*_*t*_ denotes measurement-noise with precision *σ*. The both random variables are drawn from zero-mean Gaussians: *η*_*t*_ ∼ *N*(0,(*α*ϒ_*x*_)^−1^) and *ε*_*t*_ ∼ *N*(0,(*σ*ϒ_*y*_)^−1^), where ϒ_*x*_ and ϒ_*y*_ denote their corresponding inverse covariance matrices. The state equations (Eq 1) are derived from a set of first-order nonlinear differential equations which formulate the interaction between the underlying neuronal mechanisms. By integrating these equations and passing the [Ca^2+^] states through the observer equation, *F*_*t*_ traces are generated. This operator is assumed to be a saturating (nonlinear) function.

In what follows, we use Eq 1 as the basis for all following models, where the evolution and observation functions are specified in detail to derive several models that we used to analyze calcium imaging data. One guiding principle for the following selection of biophysical models is that we prefer models with low complexity and dimensionality, i.e. a low number of parameters and variables. Theoretical considerations and preliminary analyses showed that it is unlikely that all parameters of highly detailed, complex models can be inferred from fluorescence traces, which are temporally smooth in comparison to spike data.

### Evolution functions

Evolution functions are the neuronal models which formulate the neuron biophysics; they link the dynamic change in the membrane potential (in response to input) to the [Ca^2+^] kinetics. These models are comprised of three units: (i) a spiking model which (mainly) governs the membrane potential, (ii) a model for the mechanism of HVA calcium channels where the membrane potential controls the amount of calcium current entering the neuron, and (iii) an equation to model the modulation of [Ca^2+^] due to both the removal mechanisms of free cytoplasmic Ca^2+^ and the change in calcium current. The resulting [Ca^2+^] kinetics will be used later in the observation equation in order to compute the fluorescence trace (see Eq 12).

### Spiking models

Spiking models can be categorized broadly into two groups: single- and multi-compartment models. The spiking models reported in the literature, for example [29,32–37], can reproduce a wide-range of repetitive spiking and/or bursting firing patterns of neurons; for reviews see also [20,21,38,39]. Since the fluorescence traces we considered were extracted exclusively from the somata, it is a sufficient approximation here to adopt single-compartment models of the cell body. In principle, any model based on differential equations can be used as a generative model of spikes. To show this anecdotally but also to investigate whether there is any particular model that may be best suited for modelling calcium transients, we selected three different models. We used two repetitive spiking models (i.e., the Fitzhugh-Nagumo model and Quadratic-Gaussian integrate-and-fire (I&F) model) with different model complexities (i.e., number of parameters and variables), as well as a model for compound spiking and/or bursting patterns (i.e., bursting-Quadratic-Gaussian I&F). We motivate the choice of each of these three models, in the three next sections below.

In all spiking models considered next, the total input current to neuron is *I* = *I*_*app*_ + *I*_*syn*_, where *I*_*app*_ and *I*_*syn*_ are applied and synaptic currents, respectively.

#### FitzHugh-Nagumo Model (FHN)

The FitzHugh-Nagumo model is a two-dimensional reduction of the Hodgkin-Huxley (HH) model and is one of the most widely-used models of spike generation [40–42]. Unlike the HH model, which has four dynamical variables with highly nonlinear equations, the FHN model is based only on a 2D system of equations with less nonlinearities. This reduced complexity and dimensionality motivated our choice of the FHN model, rather than the HH model. The FHN model describes the spike generation by two equations:

$$\frac{d}{dt}V=V-\frac{V_{}^{3}}{3}-W+I$$ (2)

$$\frac{d}{dt}W=\lambda \left(V+\psi_{1}-\psi_{2}W\right)$$

where parameters *λ*, *ψ*_1_ and *ψ*_2_ are constants, *V* denotes the membrane potential, and *W* denotes the recovery variable of the membrane; *V* and *W* are dimensionless variables that have biophysical roles and time scales similar to the voltage-gated ionic channels (e.g. activation of K^+^, and inactivation of Na^+^ channels) in the HH model [35]. Note that the FHN model does not fire all-or-none spikes. Depending on the input strength, it may generate subthreshold responses, or partial- or full-amplitude spikes [41]. As a consequence, similarly to the HH model, no well-defined firing threshold exists in the FHN model [41,43].

#### Quadratic-Gaussian Integrate-and-Fire model (QGIF)

For a further reduction of model complexity, we can use single-variable (i.e. 1D) spiking models instead of the 2D FHN model. The 1D models such as standard I&F model [44–47], or its analogues [48] use the membrane potential as the only dynamical variable to generate spikes. From our perspective, the main drawback of these so-called hybrid models is their non-differentiability; that is, they demand (at least) one *if* condition due to the resetting of the membrane potential whenever a spike reaches its peak. The generative models in the presence of such a step function cannot be well inverted by our VB-Laplace method. To overcome this technical difficulty, we extended a quadratic I&F model. The advantage of the quadratic I&F model is its activity-dependent threshold (for non-zero inputs) and spike latency [21], as found experimentally. In addition, it produces the upstroke of a spike by the bistability of its resting and repetitive spiking states. Therefore, this model only requires one resetting condition which is used for hyperpolarizing the membrane potential at the spike’s peak. Furthermore, it is computationally affordable [49] when compared to other nonlinear alternatives such as exponential I&F model [48]. In summary, by adapting this hybrid model [48] we created a novel 1D spiking model (i.e. the quadratic-Gaussian I&F (QGIF) model) without any discontinuous reset condition. This makes the model differentiable and invertible with the proposed Bayesian approach. The membrane potential in the QGIF model is governed by the following equations:

$$\begin{array}{l} C\frac{d}{dt}V=I-I_{L}+f_{sp}\left(V\right)-\frac{f_{rep}\left(V\right)}{\gamma }\\ I_{L}=g_{L}\left(V-E_{L}\right) \end{array}$$ (3)

where *C* denotes the membrane capacitance, *g*_*L*_ the leak conductance, *I*_*L*_ the leak current, and *E*_*L*_ the leak reversal potential. Finally, *f*_*sp*_(*V*) and *f*_*rep*_(*V*) are the functions which describe the currents relating to the spiking- and repolarization-phases of each spike, respectively. In this model we set the parameter *γ* equal to *dt* / *C*, where *dt* denotes the integration time-step size. Following [48], we defined the spike-generating function by a quadratic function:

$$f_{sp}\left(V\right)=\frac{g_{L}}{2\Delta_{th}}{\left(V-V_{th}\right)}_{}^{2}+I_{L}-I_{th}$$ (4)

where Δ_*th*_ denotes the spike slope factor, and *I*_*th*_ the threshold current which corresponds to the voltage threshold *V*_*th*_. After generating a spike, the original quadratic I&F models, for example [48,50], use a reset of *V* while we use here a repolarization function which exerts an instantaneous repolarization that lasts for one time bin *dt*; the resulting intrinsic continuity in the membrane potential renders our spiking model non-hybrid. We formulate this function as a combination of a delta-like Gaussian and a steep sigmoid function:

$$G_{rep}\left(V\right)=\frac{a_{rep}}{\sigma_{peak}\sqrt{\pi }}\exp\left(-{\left(\frac{V-V_{peak}}{\sigma_{peak}}\right)}_{}^{2}\right)$$ (5)

$$f_{rep}\left(V\right)=\frac{{\bar{I}}_{rep}}{1+\exp\left(-b_{rep}\left(G_{rep}\left(V\right)-c_{rep}\right)\right)}$$ (6)

where *a*_*rep*_, *σ*_*peak*_, *b*_*rep*_ and *c*_*rep*_ denote the generic shape parameters of these functions, *V*_*peak*_ denotes the height of the spike’s peak, and ${\bar{I}}_{rep}$ denotes the size of the following repolarization after each spike. In our parameterization for this model, the value assigned to ${\bar{I}}_{rep}$ shifts the membrane potential to more negative voltages than the resting potential *V*_*rest*_ to model the hyperpolarization-phase of the spike. The QGIF model (also its bursting version; see below) is able to exhibit reasonable neuronal responses to both constant and fluctuating (synaptic) inputs. Fig 1B shows the response of this model to a sustained applied current.

#### Bursting-Quadratic-Gaussian Integrate-and-Fire model (bursting-QGIF)

**Fig 2:**
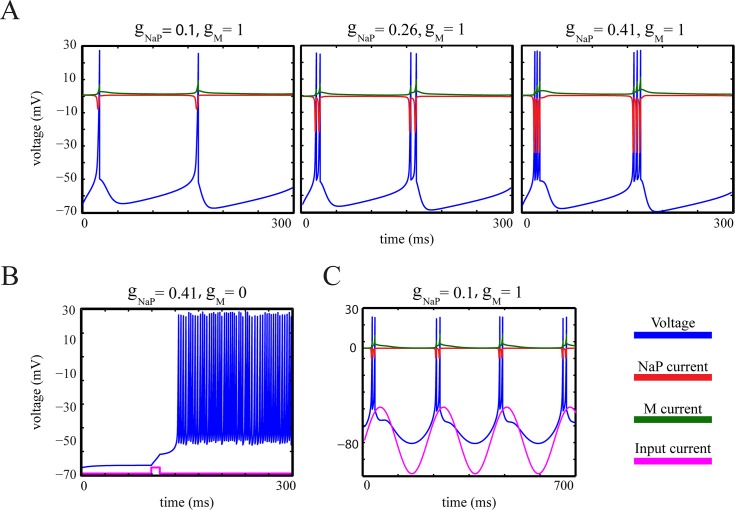
Assessment of biophysical aspects of the bursting-QGIF model. (**A**-**C**) Simulated voltage traces of the bursting-QGIF model in response to (**A**) sustained (not shown) and (**B**) brief square (magenta line) positive current pulses, and (**C**) a sinusoidal input current (magenta line). (**A**) The increment in persistent Na^+^ current enhances the burstiness, similarly to [57]: As *g*_*NaP*_ increases, the number of spikes within each burst is increased, and the interspike intervals become shorter. Note that as expected biophysically the model exhibits a tonic repetitive spiking pattern for weak *g*_*NaP*_ densities. (**B**) M-type K^+^ current governs the recovery mechanism for membrane potential, similarly to [57]: Blocking the M-type K^+^ channel by setting *g*_*M*_ = 0 leads to a prolonged burst in response to a short-duration depolarized current pulse (magenta line). This indicates that the activation of this channel is important for terminating the bursts. (**C**) The bursting-QGIF model is selective in the input slope, similarly to [53]: the periodic burst response of the model to a sinusoidal input current shows that the burst are mainly initiated on the positive slope of the input (magenta line) thus signalling the input slope. The conductances and currents across **A**-**C** are in units of [*mS* / *cm*^2^] and [*μA* / *cm*^2^], respectively; input currents are in arbitrary units. Parameters for the simulation: (**A**-**C**) ${\bar{I}}_{rep}=80\;\mu A\;mS/\mu F$, (**A**) *I*_*app*_ = 1, (**B**) *I*_*app*_ = 0.6, (**C**) *I*_*app*_ = 2.5sin(0.03*t*) *μA* / *cm*^2^. See Table 1 for the rest of parameter values.

Many neurons fire bursts of spikes, for a review see [51]. The bursts are thought to play an important role in neural information transmission [28,51–54]. These firing patterns may also be intermixed by those of repetitive single spikes [28]. In this work, we aim at the precise reconstruction of the within-burst spikes from fluorescence traces. As a representative, we will demonstrate such reconstruction for the somatic bursts of hippocampal pyramidal neurons [28–30,55]. To do this, we require a model with low complexity (for inversion) but coverage of the important biophysics of these neurons. For this purpose, we adapted the QGIF model of the previous section to derive a bursting-QGIF model by incorporating the non-inactivating muscarinic-sensitive (M-type) K^+^ and persistent Na^+^ currents (*I*_*M*_ and *I*_*NaP*_, respectively). The contribution of these currents to bursting activity of hippocampal pyramidal neurons has been already reported [56–59]. In brief, the slow activation of *I*_*M*_ during the bursting period is likely to be involved in the burst termination, and the increase of *I*_*NaP*_ can give rise to bursts as well as an increase in the number of spikes per burst. We followed [57] for both formulating and setting the parameters of these currents. Overall, the current balance equation for the bursting-QGIF model is given by:

$$C\frac{d}{dt}V=I-I_{L}-I_{NaP}-I_{M}+f_{sp}\left(V\right)-\frac{f_{rep}\left(V\right)}{\eta }$$

$$\frac{dz}{dt}=\frac{z_{\infty }-z}{75}$$

$$I_{NaP}=g_{NaP}r_{\infty }\left(V\right)\left(V-E_{Na}\right)$$ (7)

$$I_{M}=g_{M}z\left(V-E_{K}\right)$$

$$r_{\infty }=1/{\left(1+\exp\left(-\frac{V+41}{3}\right)\right)}_{}^{-1}$$

$$z_{\infty }=1/{\left(1+\exp\left(-\frac{V+39}{5}\right)\right)}_{}^{-1}$$

where *z*_∞_ and *r*_∞_ denote the steady-state activation values for gating variables of *I*_*M*_ and *I*_*NaP*_, and *E*_*K*_ and *E*_*Na*_ (resp. *g*_*M*_ and *g*_*NaP*_) denote the reversal potentials (resp. the maximal conductances) of these currents. The slow gating variable *z* is the probability of activation of *I*_*M*_. Note that the inference may assign negative values to this variable; this would lack physical meaning. In order to constrain the solution, without qualitatively changing the dynamics, we reformulate the model using a first-order Euler method, see also [60]:

$$z_{}^{aux}\left(t+dt\right)=z_{}^{aux}\left(t\right)+\frac{dt}{75}\times \frac{z_{\infty }\left(V\left(t\right)\right)-z\left(t\right)}{z\left(t\right)-z_{}^{2}\left(t\right)+O}$$

$$z\left(t\right)=1/\left(1+\exp\left(-z_{}^{aux}\left(t\right)\right)\right)$$ (8)

$$I_{M}\left(t\right)=g_{M}z\left(t\right)\left(V\left(t\right)-E_{K}\right)$$

where we expressed the dynamics of the gating variable *z* in terms of the variable *z*^*aux*^ to constrain *z* to the interval [0,1], thereby providing biophysically interpretable states for the activation of *I*_*M*_. Parameter *O* can be set properly to retain numerical stability. Furthermore, the bursting-QGIF model can account for a variety of firing patterns which were reported by previous electrophysiological experiments, for example [30,37,56,57,59]. Fig 1C shows a typical bursting voltage trace of this model when stimulated by a sustained applied current. In Fig 2, we provide a more detailed assessment of this model.

### Calcium dynamics

#### Calcium channels

Voltage-gated calcium channels have been categorized into high- and low-voltage-activated (HVA and LVA, respectively) channels [61], for a review see [1]. Unlike LVA (T-type) calcium channels, somatic calcium transients due to spikes are known to be largely mediated by HVA calcium channels (L-, N-, P/Q-, and R-type) whose activation threshold is considerably more positive than typical resting membrane potentials [5,62]. Importantly, it has been found for hippocampal CA3 neurons that L-type calcium channels are predominantly localized in somata, and have a major role in somatic calcium transients [63]. In our experiment the region of interest for extracting the fluorescence traces was also restricted to cell bodies. In addition, previous studies reported that in recorded fluorescence traces there is nearly no evidence for subthreshold fluctuations in the membrane potential [5]. Consequently, we assumed that the recorded fluorescence transients relate to the surge of Ca^2+^ through L-type channels. To model these channels, we followed [64], as the underlying mechanisms in the chosen parameter regime (see Table 1) are comparable to the Cav 1.2 and Cav 1.3 forms of the L-type calcium channels [65]. In addition, the model has less complexity as compared to other models proposed for L-type calcium channels; for a review see [66]. Overall, this model [64] formulates the calcium current, *I*_*Ca*_, across the membrane as:

$$I_{Ca}=g_{Ca}s_{\infty }\left(V-E_{Ca}\right)$$ (9)

where *g*_*Ca*_ and *E*_*Ca*_ denote the corresponding maximal conductance and reversal potential, respectively. The steady-state voltage-dependent activation of this channel, *s*_∞_, is described by the Boltzmann function (see also [64,67]):

$$s_{\infty }\left(V\right)={\left(1+\exp\left(-\left(V-V_{1/2}\right)/\rho \right)\right)}_{}^{-1}$$ (10)

which is characterized by a half-activation voltage, *V*_1/2_, and a slope factor, *ρ*. This model formulates *I*_*Ca*_ as a non-inactivating current whose activation is an instantaneous function of the membrane potential.

Eqs 9 and 10 are based on the biophysical ranges of the membrane potential. However according to the parameter regime of the FHN model (see Table 1) each full spike in this model has a peak and undershoot of 2 and -2, respectively. Therefore, the voltage will be scaled as: *ι*_1_*V*−*ι*_2_, where we set *ι*_1_ = 30 and *ι*_2_ = 40. Note that the QGIF models are already in a biologically plausible range; for example, in the QGIF model each spike has a peak around 30 *mV* followed by a hyperpolarization at around -80 *mV*.

#### [Ca^2+^] kinetics

The equation of [Ca^2+^] kinetics is given in [68] as:

$$\frac{d}{dt}[Ca_{}^{2+}]=-\kappa_{Ca}I_{Ca}-\frac{[{\text{Ca}}^{2+}]-{\left[{\text{Ca}}^{2+}\right]}_{base}}{\tau_{Ca}}$$ (11)

where the opening of the calcium channels elevates the cytosolic [Ca^2+^] using entry via *I*_*Ca*_, which subsequently decays back down to its basal concentration, [Ca^2+^]_base_, by time-constant *τ*_*Ca*_. Parameter *κ*_*Ca*_ converts the calcium current to calcium concentration (per time unit), and also scales the amplitude of the calcium transient during the activation of the channel. The negative sign of *I*_*Ca*_ renders it an inward current. Note that the decay kinetics of the calcium transient reflect the decline in [Ca^2+^] as a result of different pumping and buffering mechanisms; for a review see [1].

### The observation model

The transformation between [Ca^2+^] kinetics and fluorescence responses can be described by a saturating Hill-type function [19,69,70]:

$$g\left(\left[{\text{Ca}}^{2+}\right]\right)=F_{t}=\kappa_{F}\frac{\left[{\text{Ca}}^{2+}\right]}{[{\text{Ca}}^{2+}]+K_{d}}+d_{F}$$ (12)

where *κ*_*F*_ and *d*_*F*_ are scale and offset parameters for the fluorescence trace and parameter *K*_*d*_ is the so-called dissociation constant [26], a quantitative measure of the affinity of the fluorescent indicator to calcium.

In practice, for each fluorescence trace, we estimate the measurement SNR by dividing the minimum amplitude of the fluorescence transient by the standard deviation (std) of the baseline fluorescence, similarly to [19,69,71].

### Summary: The full generative model

In brief, each generative model (Eq 1) is a combination of the equations for a spiking model, the HVA calcium channel (Eqs 9 and 10), [Ca^2+^] kinetics (Eq 11), and the observation (Eq 12). As spiking models we use the FHN model, (Eq 2), and the family of differentiable integrate-and-fire models, for single spikes (QGIF, Eqs 3–6), and spike bursts (bursting-QGIF, Eqs 4–7), see also Table 3 for an overview. The parameter values for all models have been summarized in Table 1, or are indicated in the figure captions of the Results section below. More details are available in the S1 Appendix.

**Table 3:** Generative models.

		Spiking models		Calcium channel		[Ca^2+^] kinetics		Ca^2+^ -fluorescence mapping		Generative model’s name
Eq 1	**{**	**QGIF** (Eqs 3–6)	+	Eqs 9–10	+	Eq 11	+	Eq 12	**}** =	**QGIF**
		**Bursting-QGIF** (Eqs 4–7)								**Bursting-QGIF**
		**FHN** (Eq 2)								**FHN**

Overview of the three generative models QGIF (Quadratic-Gaussian integrate-and-fire) model, bursting-QGIF model, and Fitzhugh-Nagumo (FHN) model, and the equations required for their construction. The three models only differ in the spiking model.

### Bayesian inference

This section describes the main concepts of the VB-Laplace method, a Bayesian inference method for stochastic nonlinear state-space models [31]. The inference is based on a probabilistic generative model which quantitatively describes how observed data are generated. For a given generative model, say model *m* and data time series *y*_*t*_, the task is to infer the moments of the posterior (the so-called conditional) distributions *p*(*υ*|*y*_*t*_,*m*) for the parameters/variables of interest *υ* = {*x*_*t*_,*φ*,*θ*,*α*,*σ*} (see Eq 1), by using variational Bayes [72]. In this method the moments of the posterior distribution (conditional mean *μ* and covariance ∑) are updated iteratively by optimizing a free-energy lower bound, *F*(*q*,*y*_*t*_), on the log-evidence (i.e. the logarithm of the model evidence) with respect to an approximate posterior density, *q*(*υ*). The free energy is the difference between the Kullback-Leibler divergence (denoted by *D*_*KL*_(⋅||⋅)) of true and approximate posterior densities, and the log-evidence [72]:

$$F\left(q,y_{t}\right)=\ln p\left(y_{t}|m\right)-D_{KL}\left(q\left(\upsilon \right)\|p\left(\upsilon |y_{t},m\right)\right)$$ (13)

Variational Bayes aims at minimizing the Kullback-Leibler divergence so that the approximations to both posterior *p*(*υ*|*y*_*t*_,*m*)≈*q*(*υ*) and log-evidence ln *p*(*y*_*t*_|*m*) ≈ *F*(*q*,*υ*) become analytical (i.e. can be computed iteratively in a computationally efficient fashion). This minimization is equal to maximizing the free-energy, with respect to *q*(*υ*). Note that the divergence is a non-negative value (thus, the free-energy is a lower bound on the log-evidence), and *q*_exact_(*υ*) = *p*(*υ*|*y*_*t*_,*m*). The VB-Laplace method [31] inverts the generative model under two simplifying assumptions: (i) a mean-field separability assumption [73] which factorizes the *q*(*υ*) into the product of approximate marginal posterior densities, over the model unknown quantities (i.e. *υ*), and (ii) a Laplace approximation whereby each of these marginal densities (except those for precision-parameters) is approximated by a Gaussian density, namely $q\left(\upsilon_{i}\right)\approx N\left(\mu_{\upsilon_{i}},\sum_{\upsilon_{i}}\right)$. The first assumption facilitates the iterative maximization of free energy, and the latter finesses the analytical intractability problem of the inference; this problem arises from the nonlinearities in the likelihood (i.e. observation) functions. To update the marginal densities, the VB-Laplace method uses an iterative regularized Gauss-Newton scheme [74]. The precision-parameters are updated without requiring the Laplace approximation. Furthermore, the update rules of the hidden states exploit a variational Bayesian Laplace treatment of the extended Kalman-Rauch filter/smoother. Conceptually, given the full data time series, these probabilistic filters evaluate the approximate marginal posterior density on the hidden-states time point by time point, i.e. *q*_*i*_(*x*_*t*_|*y*_1:T_), instead of capturing the full joint density over the whole time series, i.e. *q*_*i*_(*x*_1:T_|*y*_1:T_). Therefore, the VB-Laplace method can control the lagged Kalman forward pass [31,75] by specifying to what extent this lag, *k*, is going to be applied. That is, for time *t*, this scheme approximates the lagged posterior density, *p*_*i*_(*x*_*t*_|*y*_1:*t*+*k*_,*m*), by making inference on hidden-state at the current time, i.e. *x*_*t*_, after observing all data up to time *t* + *k*, i.e. *y*_1:*t*+*k*_. This step should (in general) improve the precision and the temporal smoothness of the inference on the hidden states. For full details of the VB-Laplace method we point the interested reader to [31,60].

All Bayesian inference procedures described in this study have been implemented as Matlab (MathWorks) code in the VBA toolbox (http://mbb-team.github.io/VBA-toolbox/) developed by Jean Daunizeau and colleagues.

### Experimental methods

The experimental data recorded in our lab were six *in vitro* fluorescence traces for which simultaneous electrophysiological recordings (thus, veridical spike times) were also acquired.

### Preparation of acute brain slices

All experimental procedures were carried out with approval by the local government and complied with international and European Union norms. Experiments were performed on acute brain slices prepared from C57BL/6J mice at postnatal day (P) 3–4 (P0 –day of birth). Animals were decapitated under deep isoflurane anesthesia. The brain was removed quickly and transferred into ice-cold saline containing (in *mM*): 125 NaCl, 4 KCl, 10 glucose, 1.25 NaH_2_PO_4_, 25 NaHCO_3_, 0.5 CaCl_2_, and 2.5 MgCl_2_, bubbled with 5% CO_2_/95% O_2_ (pH = 7.4). Horizontal slices (350 *μm*) comprising the CA3 region of the hippocampus were cut on a vibratome (VT1200 S, Leica) and stored for at least 1h before use at room temperature in artificial cerebrospinal fluid (ACSF) containing (in *mM*): 125 NaCl, 4 KCl, 10 glucose, 1.25 NaH_2_PO_4_, 25 NaHCO_3_, 2 CaCl_2_, and 1 MgCl_2_, bubbled with 5% CO_2_/95% O_2_ (pH = 7.4).

### Simultaneous confocal Ca^2+^ imaging and electrophysiological recordings

For recordings, slices were placed into a submerged-type recording chamber on the stage of an Eclipse FN1 microscope (Nikon). Cells were loaded with the AM-ester of the Ca^2+^ indicator Oregon Green 488 BAPTA-1 (OGB1, 340 *μM*) by pressure-ejection (30 *s*) from a glass pipette (3–6 *M*Ω) [76]. Fluorescence traces were acquired using a 16×/0.8 NA water-immersion objective (Nikon) at a frame rate of 22.6 *Hz* using a CSU10 Nipkow-disc scanning unit (Yokogawa) and a Rolera-XR camera (QImaging) controlled by the software Streampix 5 (Norpix). Excitation light at 488 *nm* was provided by a single wavelength solid-state laser (Sapphire CDRH-LP, Coherent). Electrophysiological signals were acquired using a Multiclamp 700B amplifier, a 16-bit AD/DA board (Digidata 1440A) and the software pClamp 10.4 (Molecular Devices). Signals were low-pass filtered at 3 *kHz* and sampled at 20 *kHz*. Loose-patch (seal resistance < 1 *G*Ω) or tight-seal cell-attached recordings from cells in stratum pyramidale of the CA3 region were performed in voltage-clamp mode using borosilicate glass pipettes (8–12 *M*Ω) filled with 154 *mM* NaCl. Alexa Fluor 488 (25–75 *μM*) was frequently added for pipette visualization. Holding current was manually zeroed prior to each experiment. Brief LED light pulses were used to synchronize optical and electrophysiological signals. All experiments were performed at 32–34°C at an ACSF flow rate of ~3 ml min^-1^.

### Extraction of fluorescence traces

**Fig 3:**
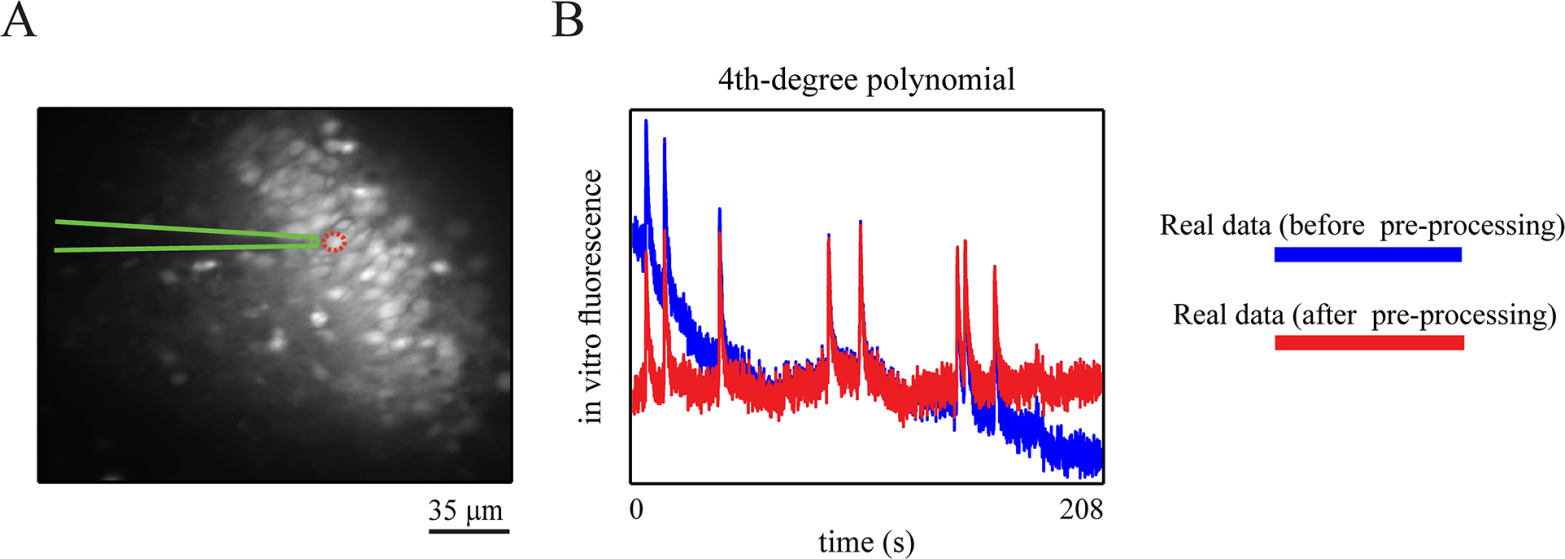
Field of view and low frequency temporal drifts. (**A**) Sample average (over frames) Oregon Green BAPTA 1 (OGB-1) fluorescence image of a neuronal population from the CA3 area of a hippocampal slice. (**B**) An *in vitro* OGB-1 fluorescence trace before (blue line) and after (red line) removal of its slowly varying components, by using the fourth degree polynomial detrending method.

We extracted the “somatic” fluorescence traces from a set of fluorescence image sequences recorded from the hippocampal CA3 neurons (see previous section); Fig 3A depicts the mean frame of one of the recorded image sequences for the whole field of view. We then converted the traces to the relative fluorescence changes (*F*_*t*_ = Δ*F* / *F*_0_) after subtraction of its resting (pre-stimulus) intensity level, *F*_0_ [77].

### Temporal drifts

A preliminary analysis of the recorded fluorescence traces (see Experimental methods) showed strong evidence of temporal low frequency drifts, which might be attributed to, for example, mechanical movements or photobleaching. In particular, we found (downward) drifts in the data lasting several hundred seconds (see Fig 3B, blue trace). This is far beyond the plausible ranges of calcium decay time-constants. Such low-frequency drifts have often been reported in time series of ECG and fMRI data, for example [78–80]. These drifts have commonly been treated as confounds, for example [81], because they can induce pronounced autocorrelation in the residual noise structures [82]. This autocorrelation may in turn decrease inference accuracy. Therefore, several methods have been suggested to remove low frequency drifts prior to analysis, for example [78,83]. Here, we decided to apply a fourth degree polynomial detrending method [78,84] to the fluorescence traces. Details for this method can be found in the S2 Appendix.

### Membrane potential thresholding

To extract the onset times of reconstructed firing events by the QGIF and FHN models (see Results), we threshold the inferred membrane potentials. For simplicity, we use as voltage threshold (which we used as spike detection threshold) the value zero. After a threshold event, we also discount any other threshold passing from negative to positive for the next 6 *ms* to prevent false spike detection from potential high frequency fluctuations in the inferred membrane potentials.

### Prior distributions

The Bayesian approach allows us to specify prior distributions to quantities of the generative model. Following [31], Gaussian prior distributions are assumed on both the evolution and observation parameters, and the initial conditions of hidden states. Each Gaussian prior is defined by its mean, *μ*, and covariance, ∑; the mean determines the prior expectation, and the covariance embodies the prior beliefs or information about the quantities of interest:

$$p\left(\theta |m\right)=N\left(\mu_{\theta }^{0},\sum_{\theta }^{0}\right)$$

$$p\left(\varphi |m\right)=N\left(\mu_{\varphi }^{0},\sum_{\varphi }^{0}\right)$$ (14)

$$p\left(x_{0}|m\right)=N\left(\mu_{x_{0}}^{0},\sum_{x_{0}}^{0}\right)$$

where the upper index 0 denotes these moments belong to prior distributions. Moreover, the form of the evolution and observation equations (note the Gaussian state- and measurement- noises in Eq 1) yields the Gaussian transition and likelihood densities, respectively:

$$p\left(x_{t+1}|x_{t},\theta ,\alpha ,m\right)=N\left(f\left(x_{t},\theta ,u_{t}\right),{\left(\alpha {ϒ}_{x}\right)}_{}^{-1}\right)$$

$$p\left(y_{t}|x_{t},\varphi ,\sigma ,m\right)=N\left(g\left(x_{t},\varphi ,u_{t}\right),{\left(\sigma {ϒ}_{y}\right)}_{}^{-1}\right)$$ (15)

Hence, the mean and variance of the prior distribution on hidden states and observations can be assigned through the definition of the evolution and observation functions, and precision quantities. In addition, Gamma priors are used on precision-parameters, where each distribution is parameterized by its shape, *a*, and rate, *b*, parameters:

$$\begin{array}{l} p\left(\alpha |m\right)=Ga\left(a_{\alpha }^{0},b_{\alpha }^{0}\right)\\ p\left(\sigma |m\right)=Ga\left(a_{\sigma }^{0},b_{\sigma }^{0}\right) \end{array}$$ (16)

### Parameters of interest

In the present models, there are five subsets of parameters: (i) calcium-dynamics-modulating parameters, (ii) membrane-potential-modulating parameters, (iii) initial conditions, (iv) observation parameters, and (v) precision-parameters. In the following, we motivate our choices for the prior distributions of these parameters. Not unexpectedly and due to the temporal smoothness of the fluorescence transients, our preliminary analyses revealed that the biophysical models are rather too complex and potentially over-fitting the data. To avoid over-parameterization, we therefore fixed several parameters to physiologically plausible values. Operationally, for a fixed parameter we set its prior variance equal to zero. This effectively prevents updating of the parameter in the VB-Laplace method. To fit the data, we kept those parameters free which influenced the kinetics of the fluorescence transients as these parameters may be inferred from the data. In the following, we will specify which parameters are fixed for the five different model components (see Table 1 for the parameter values):

(i) Calcium-dynamics-modulating parameters control the dynamics of HVA calcium channel’s gating variable and current (Eqs 9 and 10), as well as the kinetics of Ca^2+^ concentration (Eq 11). Specifically, we fixed parameters *ρ*, *V*_1/2_, *E*_*Ca*_, and *g*_*Ca*_. As free parameters, we used *τ*_*Ca*_, *κ*_*Ca*_, and [Ca^2+^]_base_. Note that as both *g*_*Ca*_ and *κ*_*Ca*_ have in Eq 11 the same effect on scaling the calcium transients we fix *g*_*Ca*_.
(ii) Membrane-potential-modulating parameters control the dynamics of membrane potential, refractoriness, and activation of the voltage-gated ion channels. Importantly, selection of inappropriate parameter regimes may preclude firing of the neuron, or trap it in a biologically meaningless state. Therefore, we fix most of these parameters in order to retain their potential physiological meanings. However, for inverting the bursting-QGIF model for data with near-millisecond resolution, we use Δ_*th*_, *g*_*M*_, and *g*_*NaP*_ as free parameters. These, in principle, can take part in shaping of spike-evoked calcium transients (see Results). In particular, by keeping *g*_*M*_ and *g*_*NaP*_ as free parameters the model will have access to a wide range of different firing regimes. It is an open and interesting question whether these parameters can be inferred from the data, e.g. at a high temporal resolution (see Results).
(iii) Prior means for initial conditions were set to their steady-state values in the absence of any input current to the neuron, i.e. when the neuron is “at rest”. We allowed the VB-Laplace method to estimate the initial values of the neuronal dynamics from the onset of fluorescence traces by keeping these as free parameters.
(iv) Observation parameters control the mapping of [Ca^2+^] kinetics on to fluorescence traces. We fix *K*_*d*_ and keep only *κ*_*F*_ and *d*_*F*_ as free parameters. We specify tighter priors on these parameters in favor of the parameters of interest listed under (i). This is because parameters *κ*_*F*_ and *κ*_*Ca*_ (and *g*_*Ca*_), as well as *K*_*d*_ all effectively scale the fluorescence transients [19,69]. Similarly, both *d*_*F*_ and [Ca^2+^]_base_ can set the offset of fluorescence trace [19]. However, our preliminary results showed that using *κ*_*F*_ and *d*_*F*_ as free parameters increased the robustness of the inference with respect to adverse conditions such as low SNR and rather variable amplitudes of the transients.
(v) Precision-parameters control the level of synaptic input to the membrane, and the noise on gating variables, recovery variable, [Ca^2+^] kinetics, and fluorescence traces. This noise level can differ, for example, among different experimental conditions. Therefore, precision-parameters need to be inferred from the data. To do so, it is sufficient to make inference about either the precision-parameters (i.e. *α* and *σ*) or the inverse covariance matrices (i.e. ϒ_*x*_ and ϒ_*y*_), see Eq 1. Following [31,60] we kept the precision-parameters free, while the inverse covariance matrices are fixed with constant values. We defined ϒ_*x*_ as a diagonal matrix and ϒ_*y*_ as a scalar whose values are constant for the whole time series.

In summary, we specify prior distributions on initial conditions, six evolution parameters *θ* = {*κ*_*Ca*_,*τ*_*Ca*_,[Ca^2+^]_base_,Δ_*th*_,*g*_*M*_,*g*_*NaP*_}, two observation parameters *φ* = {*κ*_*F*_,*d*_*F*_} and precision-parameters {*α*,*σ*}. The prior distributions are listed in Table 2. To ensure the positivity of the evolution parameters (except [Ca^2+^]_base_; see the S1 Appendix) and the observation scaling parameter (i.e. *κ*_*F*_) we re-parameterized them as $\theta_{i}=\theta_{i}^{0}\exp\left(\chi_{i}\right)$ and $\kappa_{F}=\kappa_{F}^{0}\exp\left(\chi_{\kappa_{F}}\right)$, see also [60]. That is, we estimate the posterior distribution over, e.g., parameter *θ*_*i*_ under Gaussian prior assumption on its modal parameter *χ*_*i*_; thus $p\left(\chi_{i}\right)=N\left(\mu_{\chi_{i}}^{0},v_{\chi_{i}}^{0}\right)$. $\mu_{\chi_{i}}^{0}$ and $v_{\chi_{i}}^{0}$ are the prior mean and variance of *χ*_*i*_. Accordingly, we set $\mu_{\chi_{i}}^{0}=0$, while prior knowledge about the prior expectation of the parameter will be effectively embodied in the value of $\theta_{i}^{0}$.

To assign a proper prior to calcium decay time-constant, we consider the following relationship:

$$\tau_{Ca}^{frame}\exp\left(\chi_{\tau_{Ca}}\right)\times dt_{}^{frame}=\tau_{Ca}^{real}\times dt$$ (17)

where $\tau_{Ca}^{frame}=\tau_{Ca}^{0}$ is an a-priori specified value for *τ*_*Ca*_, $\tau_{Ca}^{real}$ is the actual value of calcium decay time-constant in real time, *dt*^*frame*^ is the inverse sampling frequency (in [*ms*]), and *dt* is the integration time-step size of neuron dynamics (in [*ms*]). Eq 17 informs the generative model about the temporal precision of fluorescence data: the inversion operates on two different time scales simultaneously, a slow and fast relating to fluorescence traces (observation equation) and neural dynamics (evolution equations). The slow time scale evolves in an image frame resolution and the fast in a sub-millisecond resolution.

Note that most of the fixed parameters in both simulating and inverting tasks (Table 1), as well as the prior means for the free parameters (Table 2) were set to values according to previously reported modelling or experimental studies (e.g., see the “References” column in Table 1). See also the S1 Appendix for a more detailed explanation about our choices for a number of fixed parameters, like *V*_1/2_ and [Ca^2+^]_base_.

### Data simulation

To illustrate the method, we simulated the synthetic fluorescence traces using each of the FHN, QGIF and bursting-QGIF models, followed by inversion for each data set. For each model, the membrane was stimulated by a set of square pulses of depolarizing currents with various widths and strengths so that the spiking and/or bursting firing patterns were triggered. These traces were down-sampled at the desired frame rates (see Results). We added background noise, i.e. the trace recorded in the absence of fluorescence emission (when the laser was switched OFF), after scaling to the fluorescence traces in order to achieve the desired SNRs.

### Scaling of fluorescence data

In this study, the fluorescence traces which we used as data had a range around 0.2 (for high SNR) < max(*F*_*t*_) < 1 (for low SNR), with the baseline set to zero. For data within different ranges, one can use the priors used in this study, following by an automated normalization, see also [71], where first the baseline of the drift-corrected trace is set to zero, followed by a scaling: *F*_*t*_ ← (*scale* × *F*_*t*_ / max(*F*_*t*_)), where *scale* = 1.

### Alternative methods

We compare the spike reconstruction efficiency of our method to two different types of established spike reconstruction methods: 1) template matching [85], and 2) a deconvolution-based method [71].

For the first type, we used a widely-used template matching (TM) method using an optimally scaled template [85] implemented in the pCLAMP 10.2 software package (Molecular Devices, Sunnyvale, CA, 2009). To perform the comparisons, we use two data sets with different types of, in particular, rise kinetics (i.e. fast or slow, see Results). In an initial, interactive phase we defined a distinct template for each data set, as follows. For data with slowly rising transients, we first extracted around twenty veridical fluorescence transients from the six *in vitro* low SNR fluorescence traces, recorded in our lab. After averaging these transients, which were evoked during synchronized network activities, we found that a two-term Boltzmann equation can be well fitted to this averaged (empirical) template (see Results). In a similar way, we defined a template for the data set with fast rising transients; namely, by averaging fifty-six veridical single-spike-evoked transients extracted from twelve adopted *in vitro* traces (see below), followed by fitting a two-term Boltzmann equation to the averaged transient. Accordingly, we adopted the fitted templates by the TM method in order to detect the spiking activities in fluorescence traces. For this method a threshold (Thr_TM_) parameter for the detection-criterion needs to be set manually. In brief, this threshold embodies both the optimum scaling factor and the goodness of fit; for more detail see [85]. For each trace, we used four different thresholds as Thr_TM_ = 1, 1.5, 2 and 2.5. We then used the available joint electrophysiological and optical recordings in order to select the optimal threshold for each data set, separately (see Results). All these steps were carried out using pCLAMP 10.2.

As a deconvolution-based method, we used the fast filter technique [71] which is one the most well-established, probabilistic spike reconstruction methods. This method performs a fast nonnegative deconvolution of fluorescence traces in order to infer the most likely spike trains. It uses a first-order generative model: The calcium transients are generated through convolving the spikes, sampled from a Poisson distribution, with a decaying exponential. This model assumes an instantaneous rise time for each evoked transient, whose amplitude is linearly scaled by the number of spikes in a time frame. The generated [Ca^2+^] trace is converted to fluorescence intensities by using a linear (or saturating) mapping and adding Gaussian noise. Given the model and a fluorescence trace, the method aims to find the maximum a posteriori (MAP) spike train (the filter’s output). Note that the computed MAP is an approximation to the actual MAP. This is because for the inversion the method replaces the Poisson distribution with an exponential distribution, due to analytical intractability issues. This approximation removes the integer constraint from the number of spikes, which had been primarily determined by the Poisson distribution. Consequently, an optimal detection threshold for the filter’s output must be determined, in order to extract the best possible solution to the most likely spike train (see [71]). In other words, this thresholding is required for reporting the spike or event (single spike or burst) detection results. In our analyses, we were interested in quantifying the event, rather than individual spike detection errors (see Results). Accordingly, to make the comparison to the proposed method appropriate, when searching for an optimal threshold, we only counted the first inferred spike per event. For implementing this method we used available Matlab code (https://github.com/jovo/fast-oopsi/), with the parameter initializations performed as described in the main paper [71], including the sampling rate of the fluorescence data. More details about the method can be found in [71].

## Results

In this section, we apply the proposed method to both synthetic data and *in vitro* fluorescence measurements. In particular, we use two *in vitro* data sets, which have been acquired simultaneously with electrophysiological recordings to validate the inferred spike times: 1) a data set of six joint transmembrane current and fluorescence traces (with slowly rising transients) recorded in our lab from neonatal CA3 neurons, and 2) a data set of twelve joint membrane potential and fluorescence traces (with fast rising transients) recorded for a previous study [71] from juvenile layer V somatosensory cortex neurons (available at: https://github.com/jovo/fast-oopsi/tree/master/data/). For our approach, the values of all model parameters and their prior densities are summarized in Tables 1 and 2, unless stated otherwise in the figure captions.

For the sake of brevity, hereafter we refer to our approach (i.e. the combination of the VB-Laplace and our generative models) as “CaBBI”; an abbreviation of “*calcium* imaging analysis using *biophysical* models and *Bayesian inference*”.

### Synthetic data

In this section, we show the inversion results of the new approach for fluorescence traces simulated by the three different generative models (see Methods). More specifically, we will show that through making a reliable inference about the neuronal dynamics (like, membrane potential), our approach has the following central features:

1. It accurately reconstructs both single spikes and within-burst spikes from fluorescence transients with fast rise times (e.g. 3 *ms*). Such transients are typical for adult neurons and are the standard transient type considered in previously proposed spike/firing rate reconstruction methods, e.g. [17,19,71]; see also [86].
2. The method accurately reconstructs the single spikes from the transients even with “slow” rise kinetics (e.g. lasting 100 *ms* for a single-spike-evoked transient) as observed in the data from neonate hippocampal tissues [27], and in recordings with new genetically encoded calcium indicators [87–89]. To our knowledge, this is the first time that an (accurate) spike reconstruction approach, in practice, is applied to such data.
3. The method adapts to different kinetics of the spike-evoked fluorescence transients, for example with different rise times. This means that due to the biophysical constraints embodied by the generative model equations, the method does not require a training/conditioning or re-parameterization phase. This training step is typically performed by other methods, usually by using simultaneous optical and electrophysiological recordings, to constrain/set parameters and priors [5,13,25].
4. The method is robust against rather high levels of noise (i.e. low SNR condition). This is important because fluorescence traces with high temporal resolutions [13,90], or obtained from *in vivo* experiments [91], are usually acquired with relatively low SNR. This would mean that when using the proposed method, one can have, for example, both high temporal resolution and informative spike reconstructions.

### Simulated single-spike-evoked transients (with fast rise times)

To show that our approach can accurately reconstruct spikes from fluorescence traces, we first applied it to simulated traces resembling the single-spike-evoked transients in adult neurons with fast rise kinetics.

**Fig 4:**
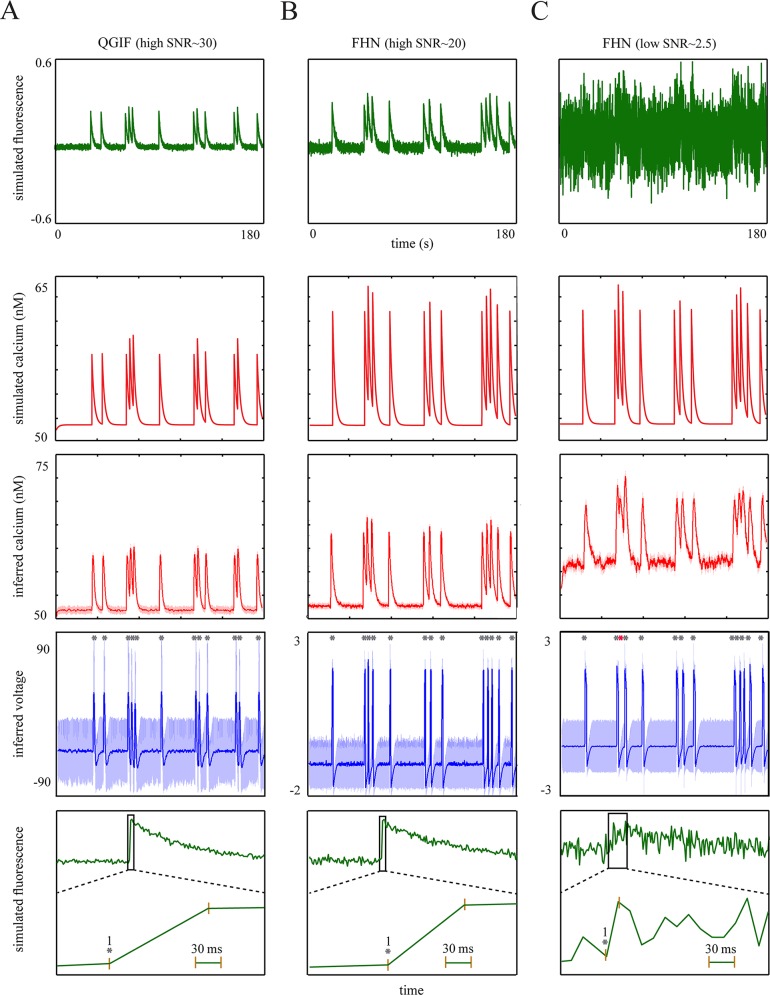
Results of the proposed approach for synthetic single-spike-evoked transients with fast rise times. (**A**): QGIF model, (**B**): FHN model with high SNR, (**C):** FHN model with low SNR. (**A**-**C**) Inferring neuronal dynamics from transients with fast rise times (e.g. 3 *ms*). The fluorescence traces (first row) and [Ca^2+^] kinetics (second row) were simulated by using (**A**) QGIF and (**B** and **C**) FHN generative models. Inverting each model for its corresponding trace by using CaBBI led to a reasonably accurate inference about the membrane potentials and non-saturating [Ca^2+^] kinetics (rows four and three); 95% confidence intervals are shown as shaded areas. The inferred spikes (fourth row) coincide accurately with the veridical spikes (grey stars). The robustness of the method against high levels of noise is evident in the results shown in **C**, where only one spike was missed (red star). Fifth row shows the zoom into the rise time of a representative fluorescence transient in each trace shown in the first row. Membrane potential has the unit of [*mV*] in the (family of) QGIF model(s), but is dimensionless in the FHN model. The fluorescence traces were sampled at a frequency of 33.3 *Hz*. The values of parameters for the simulations and inversions can be found in Table 1, and prior distributions in Table 2. Units and conventions, as well as the parameter values are consistent across the following figures, unless stated otherwise.

The generated traces are shown in Fig 4 (first row) for the two non-bursting models, QGIF and FHN. We inverted these models for fluorescence traces containing the transients with fast rise times, i.e. less than 5 *ms* (thus, imaged within one frame in the given sampling frequency of 33.3 *Hz*). The fast rise kinetics are shown in zoomed-up, representative fluorescence transients in the last row of Fig 4. This figure shows that the neuronal dynamics can be inferred reasonably accurately, including the non-saturating [Ca^2+^] kinetics (third row) and membrane potentials (fourth row). Consequently, the spikes were detected accurately, as compared to simulated spikes (grey stars in fourth row). For data with high SNR this can be seen in Fig 4A and 4B; QGIF: SNR ≈ 30, number of veridical spikes (n) = 12, missed spikes (M) = 0 and falsely detected spikes (FD) = 0, FHN: SNR ≈ 20, n = 13, M = 0 and FD = 0. The uncertainty (shaded areas) was mainly elevated for near-rest membrane potentials. This is because we modeled only HVA calcium channels, which open mainly during spikes (see Methods). We also evaluated the inference under low SNRs, see Fig 4C. These low SNRs present a lower limit of the typical SNR expected in regular experimental setups. Fig 4C (fourth panel) shows that even for this low SNR (Fig 4C, first panel), the estimated spikes match accurately the true spikes (grey stars) and only one spike was missed (red star) (Fig 4C, fourth panel; SNR ≈ 2.5, n = 13, M = 1 and FD = 0). For the low SNR case the inferred non-saturating [Ca^2+^] kinetics (Fig 4C, third panel) display a higher level of rest [Ca^2+^] as compared to the true kinetics (Fig 4C, second panel). This indicates that CaBBI has optimized the fit by estimating larger values for basal calcium concentration. For this low SNR case we used only the FHN model; qualitatively similar results can be obtained by inverting the QGIF model (not shown).

In sum, the proposed approach can reconstruct spikes veridically from single-spike-evoked transients with fast rise times, even at a low SNR level.

### Simulated single-spike-evoked transients (with slow rise times)

For slowly rising transients there is a delay (on the order of 100 *ms*) between the onset of the spike and the peak of the fluorescence transient. Although this may not be a crucial issue for reconstruction methods, this delay may cause difficulty for reconstruction methods that rely on a rather instantaneous relationship between spike and fluorescence transient peak, e.g. the sequential Monte Carlo [19] or finite rate of innovation [92] methods. Here, we show that the proposed method can precisely reconstruct single spikes even if the transients have slow rise kinetics.

**Fig 5:**
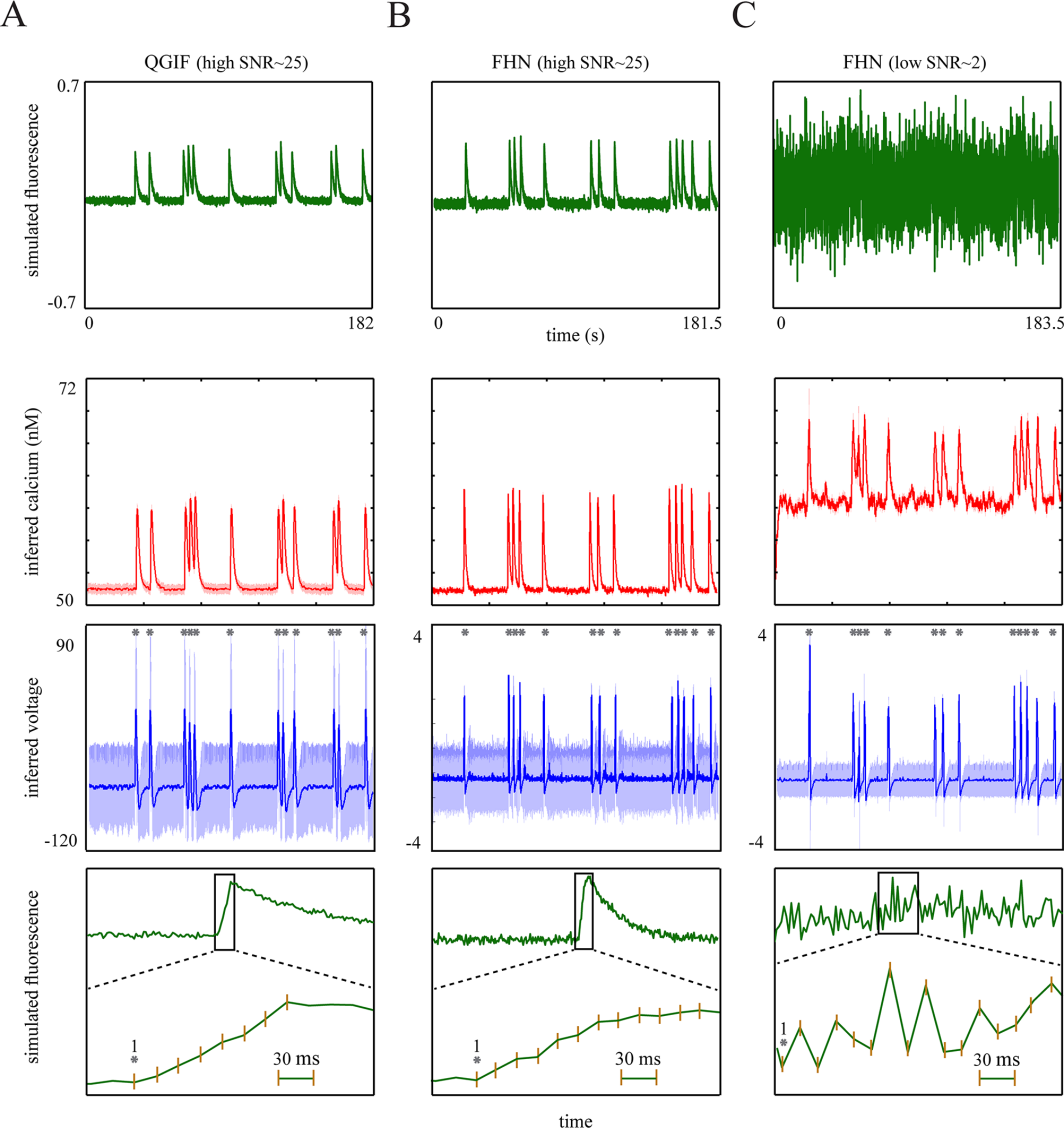
Results of the proposed approach for synthetic single-spike-evoked transients with slow rise times (around 200–450 ms). (**A**): QGIF model, (**B**): FHN model with high SNR, (**C):** FHN model with low SNR. First row: Fluorescence traces generated by each of the two non-bursting generative models. The rising kinetics of all transients were stretched using interpolation, Second row: Inferred non-saturating [Ca^2+^] kinetics, Third row: Inferred membrane potentials, where the synthetic spikes as indicated by grey stars are closely matched by the inferred spikes, Fourth row: Zoom into the interpolated, slow rise time of the first fluorescence transient in the traces shown in the first row. The time from the simulated spike (grey star) to the peak was roughly 200–450 *ms*. The decaying time-constant of the transients was about 2 seconds.

The interpolated rise kinetics of the fluorescence transients lasted around 200–450 *ms*, see the fourth row of Fig 5. We inverted the non-bursting models (i.e. QGIF and FHN) for the traces containing single-spike-evoked fluorescence transients (first row of Fig 5). The non-saturating [Ca^2+^] kinetics and veridical spikes can be accurately estimated for data with high SNR (Fig 5A and 5B), second and third rows; QGIF: SNR ≈ 25, n = 12, M = 0 and FD = 0, FHN: SNR ≈ 25, n = 13, M = 0 and FD = 0), and for the trace with low SNR (Fig 5C; FHN: SNR ≈ 2, n = 13, M = 0 and FD = 0); compare grey stars to inferred spikes in the third row of Fig 5.

We performed these reconstructions with the same parameterization as in the previous section (synthetic adult neurons with faster rise times), since our generative models do not incorporate any parameter which can explicitly capture the slow rise times in the data (*τ*_*Ca*_ corresponds only to the ‘decay’ but not the rise kinetics of the calcium transient; see Eq 11). Still, the results displayed in Fig 5 show that the models can be inverted reasonably well given data with slow rise kinetics. This is because the inference procedure takes into account the stochastic dependencies among the neuronal dynamics (see Methods), whose evolutions over time are constrained by their prior precisions. Accordingly, CaBBI adapts to different fluorescence transient kinetics (as for example here with slow rise kinetics) by using a suitable amount of state-noise on the neuronal dynamics.

### Simulated burst-evoked transients

**Fig 6:**
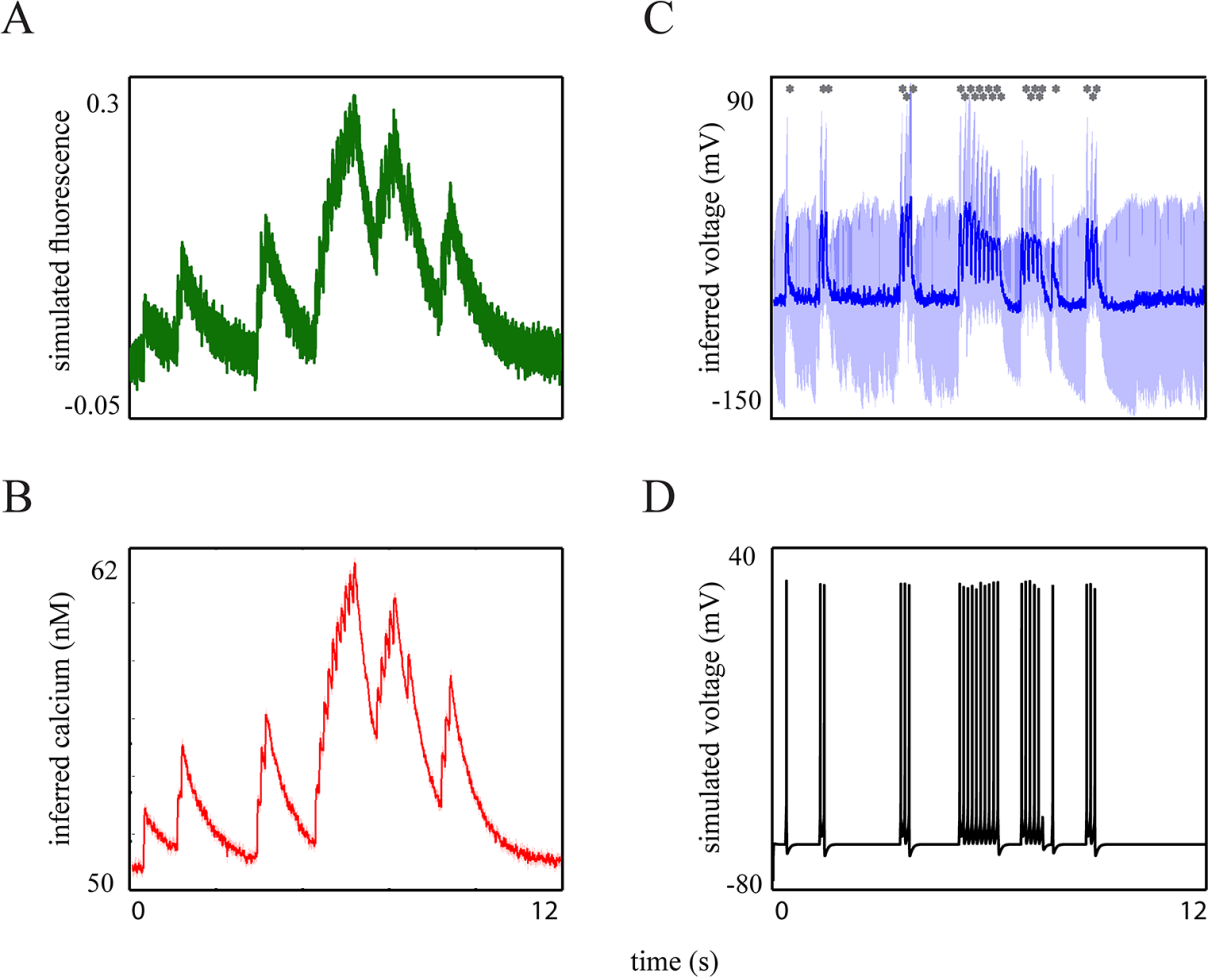
Results of the proposed approach for synthetic burst-evoked transients with fast rise times. (**A**): The fluorescence trace generated by the bursting-QGIF model, sampled at a high frequency of 700 *Hz*. (**B**): Inferred non-saturating [Ca^2+^] kinetics. (**C**): Inferred membrane potential, (**D**): Simulated membrane potential of the bursting-QGIF model. Comparison of **C** and **D** indicates that both single and within-burst spikes could accurately be detected (note also the grey stars), resulting in a precise estimation of the interburst intervals and periods of quiescence. The values of parameters for the simulations and inversions, as well as the prior distributions can be found in Tables 1 and 2.

Many neurons fire bursts, possibly intermixed with single spikes. Here we show that if the temporal resolution of the fluorescence measurements is high enough, we can still accurately reconstruct spike timing (see Fig 6). This holds true not only for single spikes but also for the spikes within a burst.

For bursts the reconstruction of spike activity from fluorescence traces becomes challenging: For high frequency and/or a high number of spikes within each burst, the impact of calcium accumulation [77] and fluorescence saturation [93,94] becomes relevant (especially when high-affinity indicators are used); in principle, these two mechanisms result in smaller spike-evoked fluorescence transients (thus, smaller effective SNR) with (probably) slower decays [19,26]. These strong nonlinearities will make the spike reconstruction challenging mainly due to the less differentiable transients during bursts.

In general, fluorescence traces with low temporal resolution, e.g. acquired at 4.2 *Hz* [95], cannot resolve the single transients evoked by each distinct spike within a burst [17,95,96]. However, recent developments in calcium imaging have made it possible to obtain fluorescence measurements at very high sampling rates such as 1 *kHz*, for example [13,25]. Assuming a sampling rate of 700 *Hz*, we generated a synthetic fluorescence trace (Fig 6A) using the bursting-QGIF model. To generate complex burst patterns (Fig 6D), we used doublet, triplet and multiplet (5 and 10 spikes) bursts, and interspersed single spikes (indicated by grey stars in Fig 6C). Although we imposed relatively high noise on the trace, all of single spikes and the within-burst spikes were inferred accurately (Fig 6C; SNR ≈ 3, bursting-QGIF: n = 25, M = 0 and FD = 0). In addition, the durations of the bursts’ active phase and the interburst intervals, as well as the periods of quiescence were reasonably well estimated (compare the simulated and inferred membrane potentials in Fig 6C and 6D). In addition and as shown in Fig 6C, for each burst the reconstructed spikes have correctly inferred incomplete repolarizations and are located on top of a plateau; this is a common bursting characteristic of many neurons, including hippocampal cells [28,29,58]. The non-saturating [Ca^2+^] kinetics can also be accurately inferred (Fig 6B) from these saturated traces with highly accumulated transients (Fig 6A). In summary, we found that the method can read out spikes within bursts observed in synthetic fluorescence traces (with fast rise kinetics) when the temporal measurement resolution is high enough, e.g. 700 *Hz*.

### Biological data

#### In vitro recorded fluorescence transients (with slow rise times)

Having accurately reconstructed spikes from synthetic data, we now apply the proposed approach to the experimental data recorded in our lab from neonate hippocampal tissue (i.e. first data set; see above). An exclusive feature of these data is the slow rise time (up to several hundred milliseconds) of fluorescence transients, consistent with previous work [27]. For model inversion based on these data, we use the same values of the parameters as for both synthetic data sets with fast rise time and slow rise time; namely, independent of our *in vitro* data. As shown above on synthetic data with slow rise time, we expect that the method can intrinsically adapt to the *in vitro* fluorescence transients without requiring any training/conditioning phase, unlike [5,13,25].

For the analysis, we used six *in vitro* fluorescence image sequences of CA3 immature tissues, as well as the simultaneously recorded transmembrane current signals recorded from two (one per image sequence) patched cells in our experiment (see Experimental methods). For these two cells, the veridical spike times provided by the electrophysiological recordings enabled us to validate the inferred onset times of spiking events (i.e. burst or single spike).

A hallmark of the measured data is spontaneous, synchronized network activities which are expressed as so-called giant depolarizing potentials (GDPs) in individual neurons [97]. In the CA3 hippocampal region, each GDP is usually seen as a slow wave-like propagation (e.g., lasting 200 *ms*) of cellular activation traveling across neurons in the direction, for example, from the CA3c area to the CA3b area [27,98]. An important variable of GDPs is the onset times of cellular activation during GDPs. If one knew the precise onset times of neurons participating in a GDP, one can analyze the underlying spatiotemporal characteristics of GDPs, i.e. the GDP’s propagation direction or velocity across the network, the GDP initiators cells, or any systematic cellular activation orders across multiple GDPs [27]. The reconstruction of such network characteristics would enable an improved analysis of the development of immature neuronal circuits, as compared to a GDP analysis based on the smeared fluorescence image sequences [27,99].

We observed the GDP-mediated spiking activity of individual neurons as single or burst of spikes, consistent with [97,100]. In the previous section, we showed that for transients of adult neurons with a fast rise time (e.g. 3 *ms*) and acquired at a high enough temporal resolution (700 *Hz*), the within-burst spikes can be reconstructed accurately using fluorescence measurements. For neonatal neurons, the rise time of each single-spike-evoked transient lasts much longer, e.g. 100 *ms* [27]. This long rise time may be caused by strongly nonlinear mechanisms of Ca^2+^ pumping and buffering [101,102]. In addition, GDP-mediated spiking events occur during synaptically-driven long-lasting depolarizations (up to several hundred milliseconds) with large amplitudes (e.g. -10 *mV*, thus mostly above the half-activation of HVA calcium channels) [97], thereby modulating the Ca^2+^ kinetics with added, pronounced nonlinearities. Effectively, for our neonatal neuronal data acquired at 22.6 *Hz*, we could not resolve the GDP-mediated within-burst spikes. This means that in the recorded fluorescence traces, both GDP-mediated burst-evoked and GDP-mediated single-spike-evoked transients were characterized by a single rising phase and a single decaying phase.

In all of these recorded fluorescence traces, there was evidence of slowly varying drifts. To ensure reliable estimates of biophysical parameters and states, we removed these drifts from the traces by using a fourth degree polynomial detrending method (see Methods). In Fig 3B, the original (blue line) and drift-corrected (red line) versions of one of the recorded fluorescence traces are plotted. In addition, we emulated the measurements at a low SNR level by scaling and adding the background noise to the fluorescence traces (see Methods).

**Fig 7:**
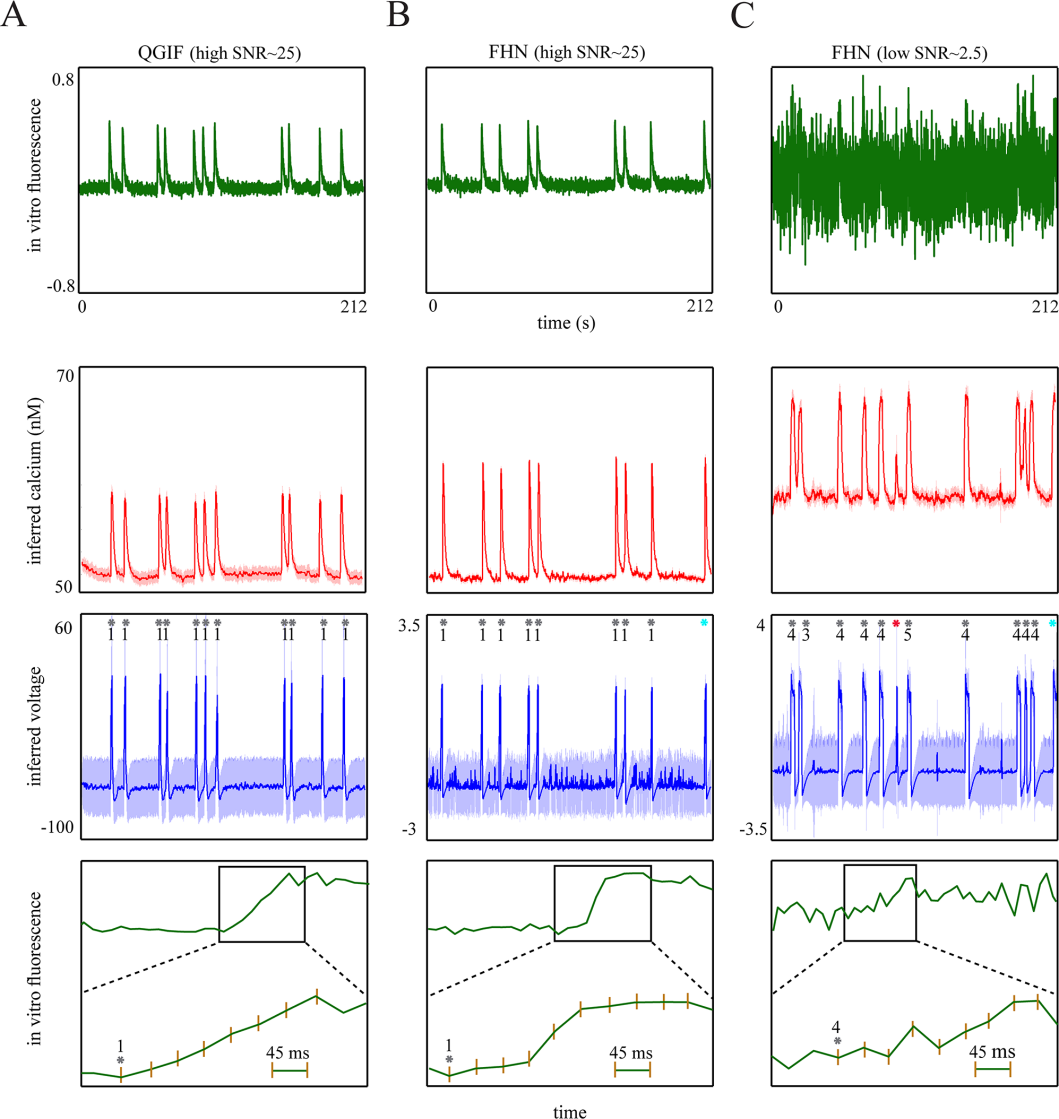
Results of the proposed approach for *in vitro* fluorescence traces with slowly rising transients (around 400 ms). (**A**): QGIF model, (**B**): FHN model with high SNR, (**C)** FHN model with low SNR. First row (**A**-**C**): The *in vitro* fluorescence traces containing transients with relatively variable and slow rise times, mediated by spontaneous GDPs. The low SNR trace in **C** was generated by contaminating the *in vitro* trace by background noise. Second row (**A**-**C**): Inferred non-saturating [Ca^2+^] kinetics, Third row (**A**-**C**): Inferred membrane potentials, where the onset times of GDP-mediated events (single spike or burst) determined by electrophysiological recordings (grey stars) and inferred spikes are highly concurrent. The two light blue stars indicate that there was no recorded transmembrane current available for the observed events in the fluorescence trace(s). Note that for a GDP-mediated burst event the onset time refers to the occurrence of its first spike. Fourth row (**A**-**C**): Zoom into the slow rise time of the first fluorescence transient in the traces shown in the first row. The numbers and stars indicate the veridical spike count and the onset time of each GDP. The rise time from GDP onsets to the fluorescence transient peaks was around 300–450 *ms*. The decay kinetics of the transients was lasting around 3.5–4.5 seconds.

**Fig 8:**
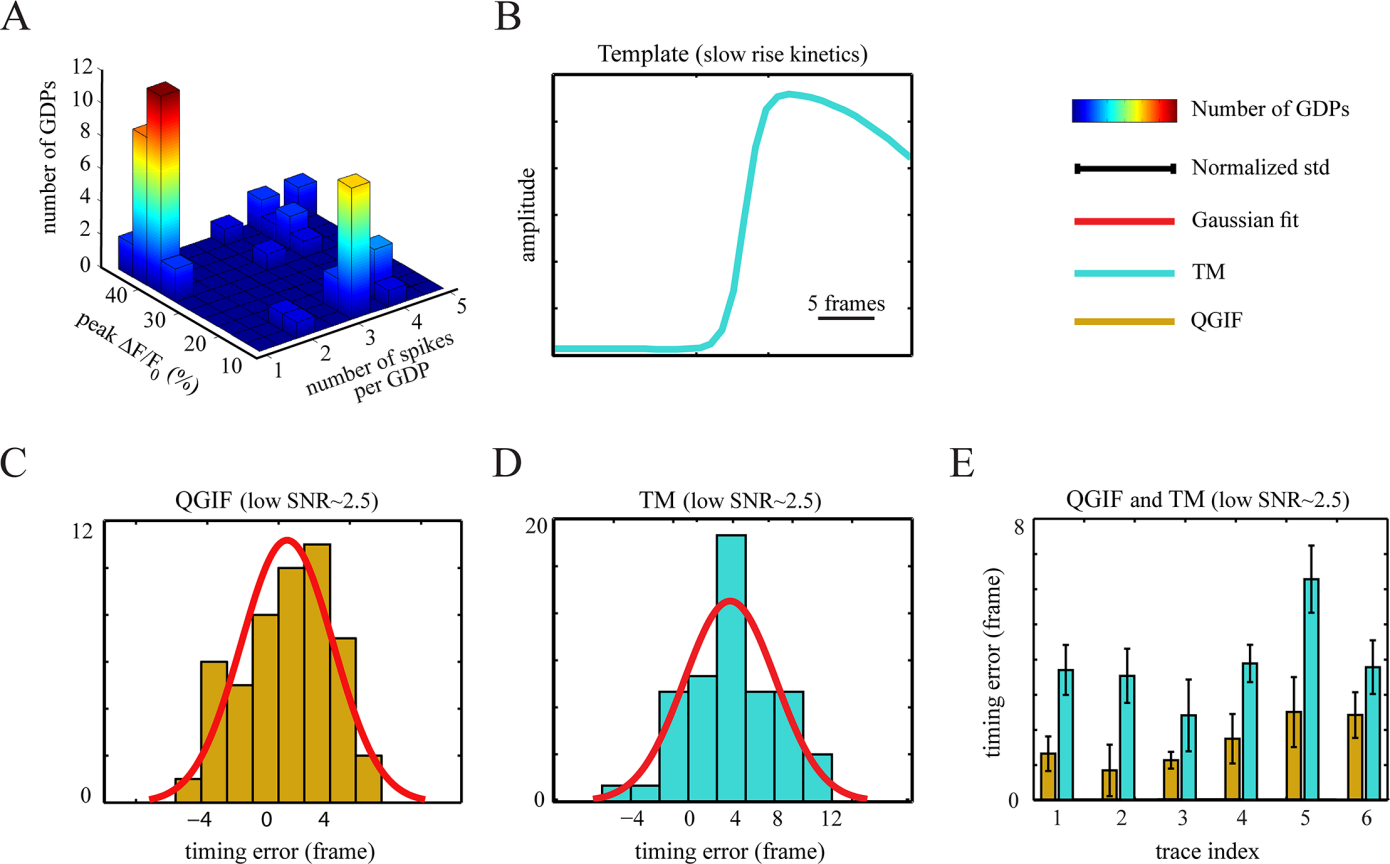
Accuracy of the reconstructed GDP onset times of the proposed approach and template matching method on *in vitro* data. (**A**) The 3D histogram shows the relation between the number of spikes per spontaneous GDPs and the peak of the fluorescence transients (n = 51 GDPs). (**B**) The two-term Boltzmann template for the template matching (TM) method derived from the first experimental data set. (**C**) Timing error distributions computed for the QGIF model inversion for all six fluorescence traces, under low SNR. Gaussian fit: mean = 1.7 and std = 3.2 time bins (i.e. frames). (**D**) Timing error distribution for the TM method, under low SNR. Gaussian fit: mean = 3.7 and std = 3.8 time bins. (**E**) Timing errors estimated by the QGIF model and the TM method plotted for each *in vitro* trace of the first data set.

In Fig 7 (see also S1 Fig) the precise detection of GDPs is shown for three representative samples (out of six) of drift-corrected *in vitro* fluorescence traces (with slowly rising transients). Critically, as mentioned above, both GDP-mediated bursts and single spikes had similar transient kinetics with a single prolonged rise (up to 400 *ms*, see fourth row of Fig 7A–7C) and large amplitudes which were rather independent of spike count (see Fig 8A); unlike the data from adult neurons [9,103].

Accepting that one cannot differentiate between GDP-mediated single-spike- and burst-evoked transients, we used the two repetitive spiking models (QGIF and FHN) to infer the onset times of individual neurons’ firing (single spike or burst) during the synchronous events. We found that both models can accurately detect the experimentally observed GDP-mediated spiking events under high SNR condition (Fig 7A and 7B), third row; QGIF: SNR ≈ 25, n = 11, M = 0 and FD = 0, FHN: SNR ≈ 25, n = 8, M = 0 and FD = 0). The inferred spike-evoked calcium transients have amplitudes mostly around 10 *nM*, consistent with [13,104]. These values were inferred from the data as we used rather uninformative priors (see Table 2). We also evaluated the performance of our approach for experimental fluorescence traces with low SNR using the FHN model (Fig 7C). The results (Fig 7C) indicate that the inversion can perform reliable inference even under low SNR: A single noise peak was miss-interpreted as a spike (third row, red star; SNR ≈ 2.5, n = 10, M = 0 and FD = 1). Although fluorescence measurements are commonly recorded with higher SNRs than the trace displayed in Fig 7C (first panel), this robustness against a high level of noise is important: In principle, for GDP experiments one may use faster cameras and scanners which will result in a lower SNR but an increased temporal resolution. Using the proposed method in combination with such fast imaging techniques, one may be able to better extract the differences in cellular activation latencies during GDP events.

### Quantification of detection accuracy: Comparisons

Here, we compare the detection accuracy of our method, CaBBI, to two well-established, widely-used spike reconstruction methods: a template-matching method [85], and a deconvolution-based fast filter method [71].

#### Comparison on first data set

CaBBI: We first quantified the GDP detection errors (Table 4) as well as the accuracy of the inferred onset times of GDPs (Fig 8C–8E) for our method, under both low SNR (~2.5) and high SNR (~25) conditions. We inverted both the QGIF and FHN models for all six drift-corrected fluorescence traces of the first data set acquired with simultaneous electrophysiological recordings. To read out the GDPs’ onset times from the inferred spikes we extracted the time points at which the membrane potential crossed the detection threshold of zero (see Methods). Both the QGIF and FHN models detected the GDPs without error under a high SNR (n = 51, M = 0 and FD = 0). There were only few detection errors under low SNR (see Table 4).

**Table 4:** GDP detection results of three different methods (first data set).

Method		# Missed GDPs	# Falsely Detected GDPs
**CaBBI**	FHN	0	1
	QGIF	2	1
**Template matching**	Thr_TM_ = 1	0	74
	Thr_TM_ = 1.5	0	1
	Thr_TM_ = 2	3	2
	Thr_TM_ = 2.5	18	0
**Fast filter**	Thr_ff_ = 0.65	16	19
	Thr_ff_ = 0.7	21	8
	Thr_ff_ = 0.75	26	7

GDP detection results of the proposed approach (CaBBI), the template matching (TM) method, and the fast filter technique. For the proposed approach we used both the FHN and QGIF models, for the TM method we employed four detection-criterion thresholds (Thr_TM_), and for the fast filter three event detection thresholds (Thr_ff_). The methods were applied to the first data set (*in vitro* data with slowly rising transients) under a low SNR (~2.5). The total number of veridical GDPs was 51. The comparison shows that both the proposed approach and the TM method have a high GDP detection accuracy, while the fast filter cannot solve this task as it was developed for transients with fast rise times.

Template matching: In an initial phase, the template (Fig 8B) was determined empirically from the first data set (see Methods). As is common practice, we used several detection-criterion thresholds and selected the best threshold manually. For the optimal threshold (Thr_TM_ = 1.5), the GDP detection accuracy of the TM method was as good as that of CABBI when using the FHN model (Table 4).

Fast filter: We applied the fast filter to the first data set under low SNR, and thresholded its outputs by using a set of event detection thresholds (see Methods). Its detection results for three different thresholds are shown in Table 4; including Thr_ff_ = 0.7 which extracted the events with relatively smaller errors (n = 51, M = 21 and FD = 8) than all other thresholds (i.e. Thr_ff_ = 0, 0.05, …, 1). Not surprisingly, this method could not perform a reliable GDP detection from these data, as it has been originally developed for deconvolving fast rising transients [71].

We also computed the ‘timing errors’ as the difference between the inferred onset times and the veridical GDP-mediated spike times measured by the simultaneously recorded transmembrane current signals (Fig 8C–8E). For GDP-mediated burst events we compared the inferred onset time to the veridical timing of the first spike of that burst. For 51 veridical GDP events the QGIF model showed a reasonably accurate inference about the onset times with a timing error of 1.7 ± 3.2 (mean ± std) for low (Fig 8C) and -0.5 ± 2.0 time bins (frames) for high SNR conditions (not shown). We found the FHN model to be clearly less accurate (timing error of 10.0 ± 10.3 time bins) than the QGIF model. We compared the timing errors of our method only to the TM method, as the fast filter technique showed an insufficient GDP detection accuracy for these data. For the optimal detection-criterion threshold of the TM method (i.e. Thr_TM_ = 1.5; see Table 4), the timing error was 3.7 ± 3.8 time bins (Fig 8D), which is significantly higher (paired-sample Wilcoxon signed-rank test (two-sided); p < 0.001) than the error of the QGIF model (Fig 8C). We also show this difference between the two reconstruction methods for single fluorescence traces in Fig 8E.

Overall, these results (Table 4 and Fig 8) indicate that the new method, CaBBI, can detect GDPs as accurately as the TM method and reconstruct the onset times more accurately than the TM method, for low SNR. In addition, CaBBI does not require an initial, interactive phase where the template is estimated and the detection-criterion threshold is selected manually.

Note that the good results of the TM method in comparison to CaBBI and the fast filter method should be interpreted with some caution. The TM method had two specific advantages not harnessed by the other two methods: 1) the fluorescence transients used to define the empirical template were extracted veridically, by harnessing the simultaneously measured electrophysiological data. In addition, the optimal detection-criterion threshold was verified through the electrophysiological data. A fairer comparison would have been to not use the electrophysiological data at all. However, this would have made the comparison operator-dependent when defining the template. 2) The TM template was derived from the same data set, for which spikes/events were detected with that template. In other words, the same data was used for ‘training’ the model, and again for spike/event detection (testing). In contrast, with CaBBI, we analyzed the data without using any prior information from the same data.

#### Comparison on second data set

To perform a comparison using data with fast rising transients, for which the fast filter technique was developed, we used twelve *in vitro* fluorescence traces (second data set, see above). These data have been used originally to evaluate the fast filter technique [71]. We performed this comparison in terms of the event, rather than individual spike, detection accuracy (see Methods), since our method was not able to reconstruct the within-burst spikes from these data (see below). In the following, we explain the comparison results for all three methods.

**Fig 9:**
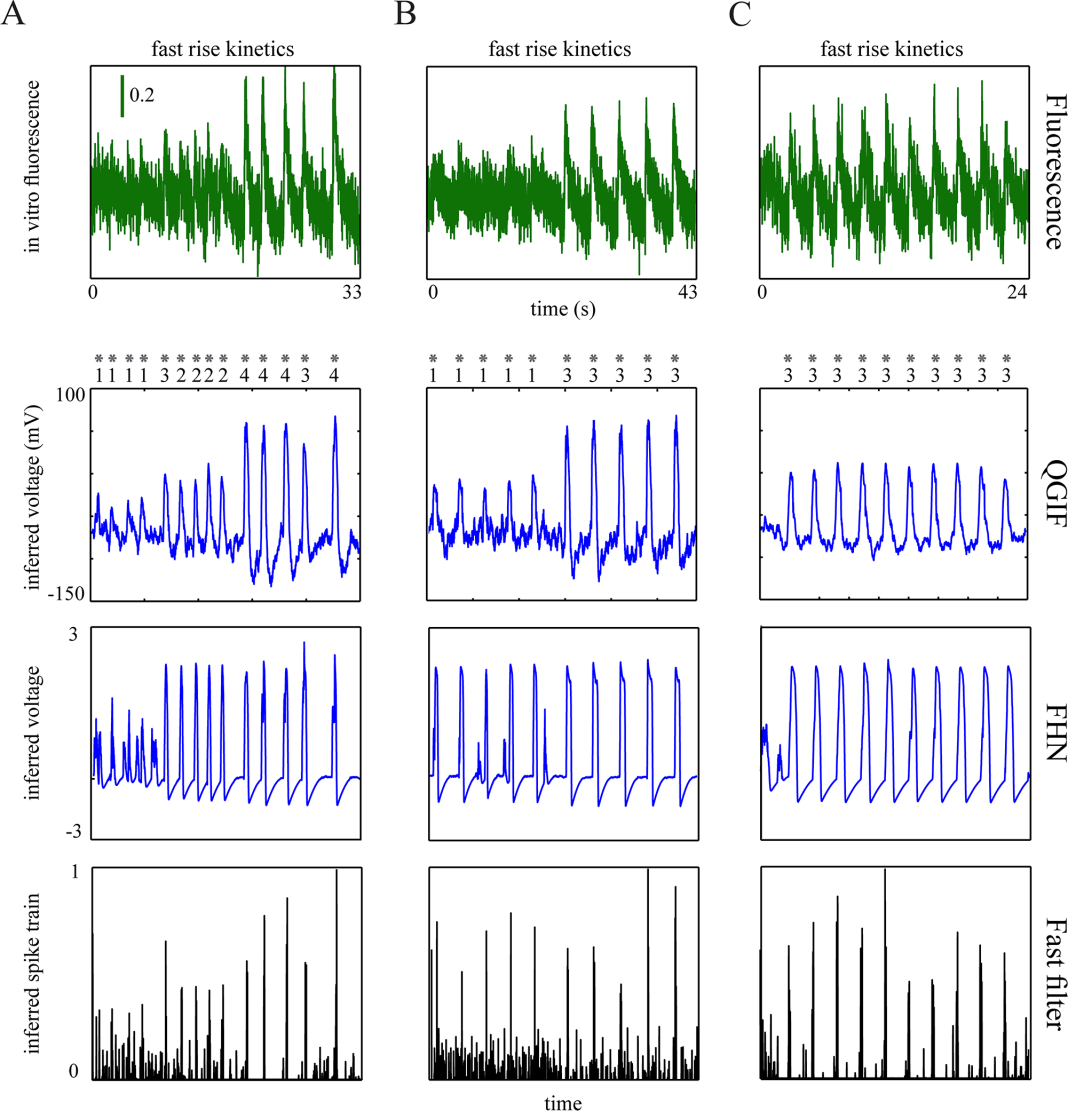
Comparison of the proposed approach and the fast filter for *in vitro* fluorescence traces with fast rising transients. First row: The *in vitro* fluorescence traces containing transients with fast rise times. Second and third rows: Inferred membrane potentials (posterior means) using CaBBI with the QGIF and FHN models. Fourth row: Inferred spike trains of the fast filter. The comparison of the veridical spiking events (grey stars) to the inferred ones (**A**-**C**, the last three rows) shows that both methods have similar event detection results for fast rising transients. The numbers below the grey stars indicate the spike count per each veridical event, determined by electrophysiological recordings.

CaBBI and fast filter: For our method, we used the same parameterization as for the first comparison, after setting *scale* = 1 (see Methods) for all twelve traces. The inversion results of our method (both the QGIF and FHN models) and the fast filter for three representative samples are shown in Fig 9A–9C. The results for these samples show that both methods have qualitatively similar, reasonably good detection accuracy of the spiking events (i.e. bursts or single spikes); note the veridical timing (grey star) and spike count per event on top of the second row, determined by the simultaneous electrophysiological recordings. Note that CaBBI inferred all the single-spike- or burst-evoked transients as if always evoked by single spikes, since the transients evoked by individual spikes are poorly resolved in these data (see [71]). In contrast to the GDP-evoked transients (see Fig 7, first row), in these data the higher spike count per event was mostly encoded by the transients with higher amplitudes (Fig 9A–9C, first row). Although hard to see from the fourth row of Fig 9A–9C, the fast filter used this feature of the data to partially infer the spikes within the burst events, when using a manually determined detection threshold (see Methods). For CaBBI, we observed that as the spike counts of events and therefore the amplitudes of the transients became more variable in a fluorescence trace (compare Fig 9A–9C, first row), inferring the events from transients with lower amplitude became more difficult. As a result, some of them were inferred as partial spikes. For instance, see the fluorescence trace in Fig 9A, which was a hard case also for the fast filter to invert.

In summary, for all twelve traces (Table 5) the FHN model had an event detection accuracy (n = 149, M = 10, and FD = 20) similar to the fast filter with the optimal threshold of 0.35 (n = 149, M = 11, and FD = 15). The QGIF model had, however, an inflated error of missed events and at the same time a considerably smaller error of falsely detected events (n = 149, M = 45, and FD = 2), as compared to the fast filter. When applying the template matching method, we first defined the empirical template, applied it to all twelve traces, and then selected the optimal detection-criterion threshold manually. Among the three methods, TM detected the events with the highest accuracy (n = 149, M = 7, and FD = 1), when using its optimal threshold which was Thr_TM_ = 1.5 (see Table 5). Although, note that the same two caveats for the TM method apply as in the comparison on the first data set (see above).

**Table 5:** Event detection results of three different methods (second data set).

Method		# Missed events	# Falsely Detected event
**CaBBI**	FHN	10	20
	QGIF	45	2
**Template matching**	Thr_TM_ = 1	0	14
	Thr_TM_ = 1.5	7	1
	Thr_TM_ = 2	32	0
	Thr_TM_ = 2.5	53	0
**Fast filter**	Thr_ff_ = 0.3	3	40
	Thr_ff_ = 0.35	11	15
	Thr_ff_ = 0.4	19	10

Event detection results of the proposed approach (CaBBI), the template matching (TM) method, and the fast filter technique. This table uses the same format as Table 4. The methods were applied to the second *in vitro* data set with low SNR fast rising transients. The total number of veridical events was 149. The comparison shows that the TM method was the best method for the optimal detection-criterion threshold (Thr_TM_ = 1.5). CaBBI (the FHN model) and the fast filter (Thr_ff_ = 0.35) had similar event detection accuracy for these data.

### Inference on GDP propagation patterns

Above, we already showed the reasonably high accuracy of our method in reconstructing GDP onset times (see Fig 8). We now show the usefulness of this accuracy in inferring the propagation patterns of GDPs, as the complex characteristics of immature neuronal circuits.

**Fig 10:**
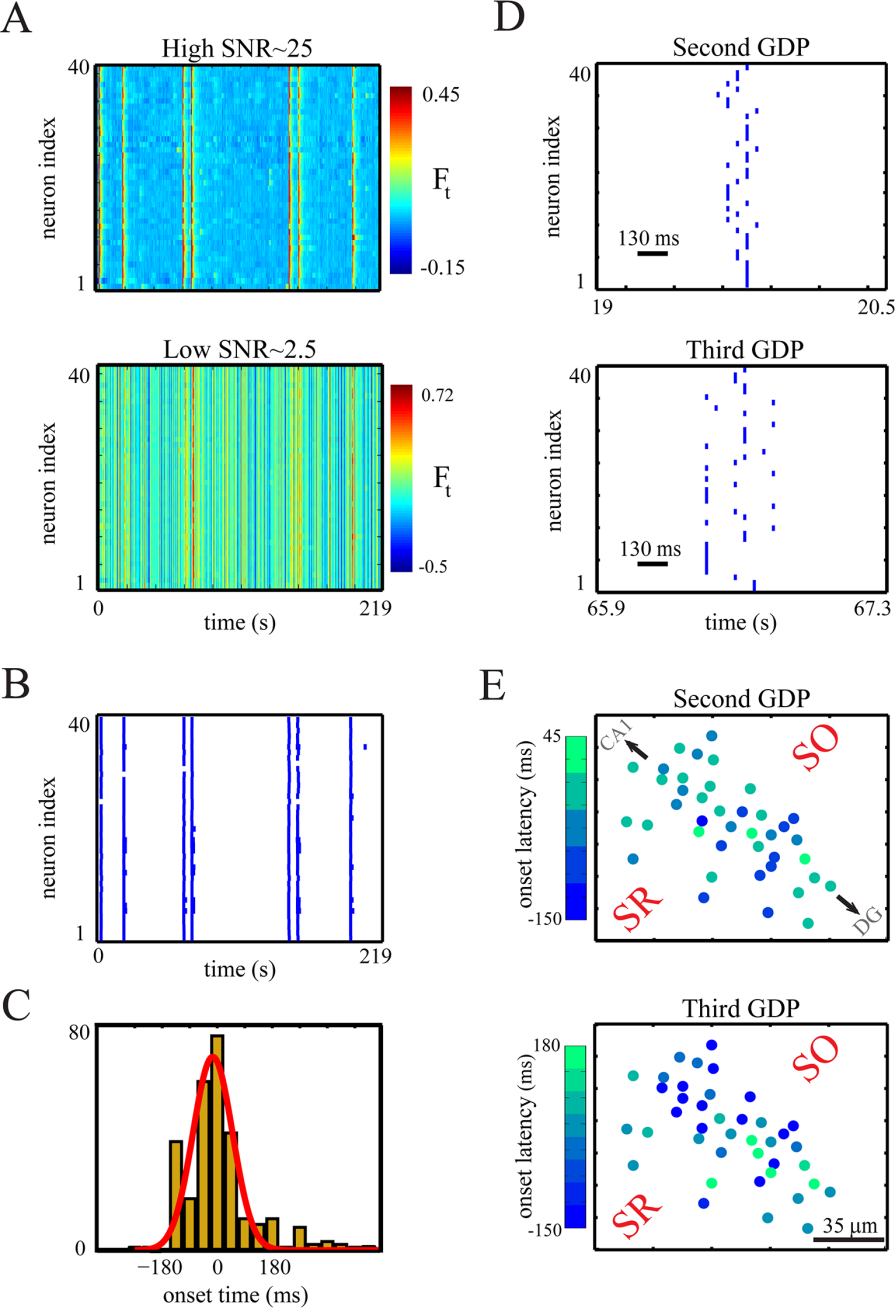
Using the proposed approach for large-scale reconstruction of the cellular activation latencies during GDPs. (**A**) Two raster plots of color-coded *in vitro* fluorescence traces for 40 CA3 immature neurons. Upper Panel: high SNR condition, Lower panel: low SNR condition. (**B)** The reconstructed patterns of onset times of spontaneous activities (both isolated and GDP-mediated events), identified by inverting the QGIF model for low SNR data (lower panel of **A**). (**C**) Histogram of cellular activation latency distributions over all reconstructed GDPs and neurons. The red curve shows a Gaussian fit: with mean = -14.5 and std = 90 *ms*. (**D**) Closer inspection of the reconstructed raster plots in B around the second and third GDPs, unveiling the cellular activation latencies during each GDP. (**E**) The spatiotemporal GDP propagation across neurons can be observed based on color-coded latencies of neurons during the second and third GDPs. SR and SO denote stratum radiatum and stratum oriens of the hippocampal CA3, and DG denotes dentate gyrus.

We illustrate the reconstruction of GDP characteristics using one representative fluorescent image sequence (out of six) recorded in our experiment. For this image sequence, the fluorescence traces were extracted from 40 well identifiable neurons. The raster plot of color-coded high SNR fluorescence traces in Fig 10A (upper panel) shows clearly visible, spontaneous synchronous network events (i.e. GDPs). The same data under low SNR condition (around 2.5; after contaminating the traces by scaled background noise; see above) is shown in the lower panel of Fig 10A. Using the new method and as a proof of principle, we reconstructed the onset times of all 40 neurons during GDPs, under the low SNR condition. For inversions, we used the QGIF model because of its lower timing error in extraction of GDPs’ onset times, as compared to the FHN model and the TM method (see above). The reconstructed events are shown in Fig 10B, where it can easily be seen that their timings show a close match to the large fluorescence changes in Fig 10A (upper panel).

The histogram of onset latencies of neurons over all GDPs is plotted in Fig 10C. For each GDP, the latencies were computed with respect to the median onset time of neurons during that GDP. In this histogram, the medians are centered on 0 *ms* so that earlier and later activations of neurons have negative and positive values, respectively. The observed latency distribution can be approximated by a Gaussian distribution with a mean of -14.5 *ms* and standard deviation of 90 *ms*, which is consistent with previous reports for the developing hippocampus [27]. For visualization of the difference between onset times, we plotted the reconstructed onset times of the second and third GDPs in Fig 10D. In addition, in Fig 10E we show the inferred onset latencies for these two representative GDPs color-coded at their actual spatial positions in the field of view. In the first of these images (second GDP), the GDP pattern starts roughly in the center of the image and spreads to both left-upward and bottom-right, i.e. from the stratum pyramidale (SP) in CA3 towards both CA1 and dentate gyrus (DG). The second image (third GDP) shows, instead, a rather clear unidirectional orientation towards DG. Such wave-like patterns of propagation of these two illustrative GDPs are consistent with previous reports [27,105].

### Inferring biophysical parameters

In this section, we show how our approach can be used for quantifying biophysically interpretable parameter changes based on calcium imaging data, e.g. for inferring changes of parameters due to pharmacological interventions. To provide a proof-of-concept, we used two synthetic data sets where we changed: 1) the calcium decay time-constant and 2) the conductance of M-type K^+^ channels.

#### Change of calcium decay time-constant

**Fig 11:**
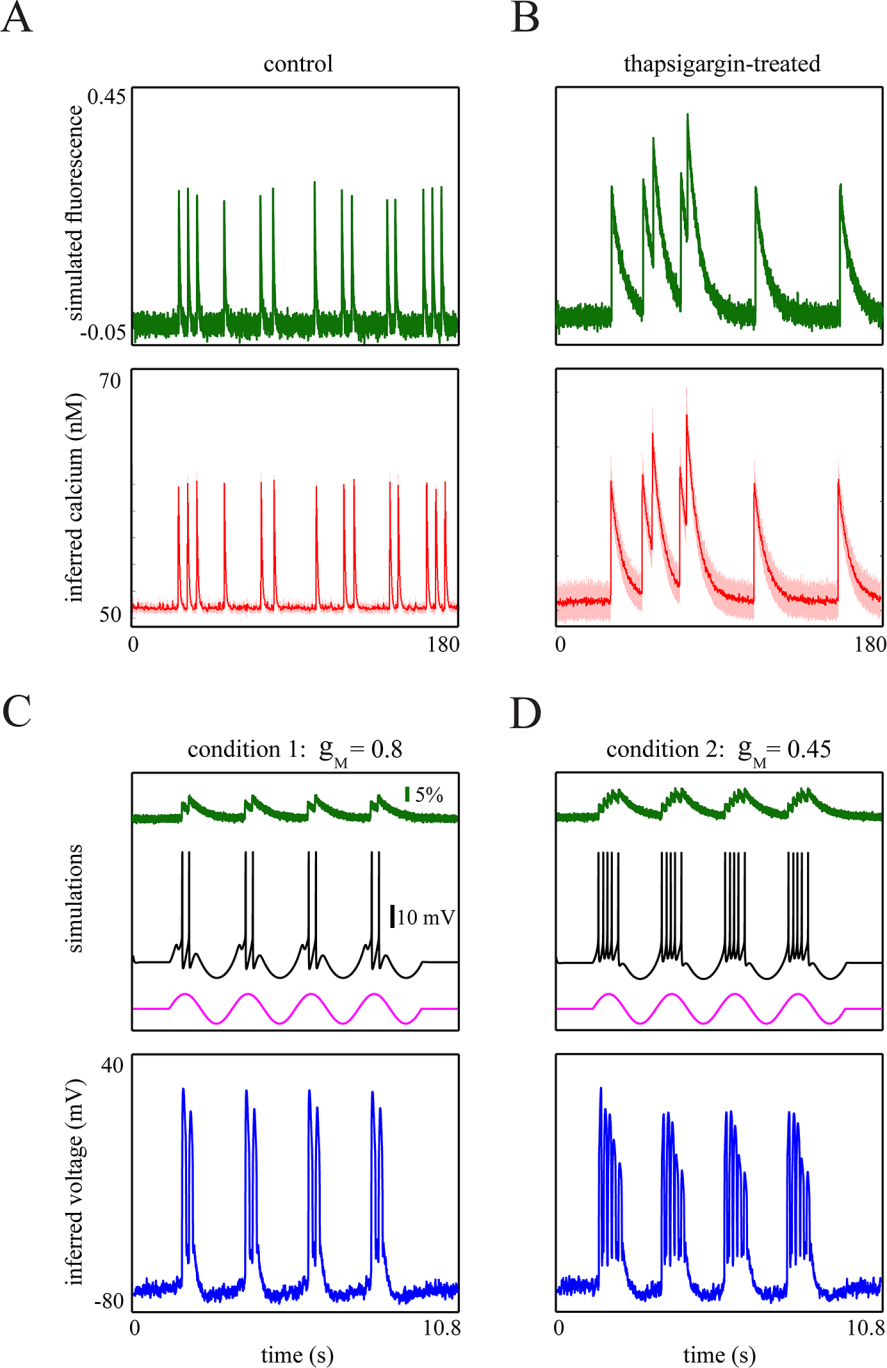
Inference about parameter changes in synthetic data. (**A** and **B**): The simulated fluorescence traces (green line) by the FHN model for (**A**) *τ*_*Ca*_ = 600 *ms* and (**B**) *τ*_*Ca*_ = 6000 *ms*. Red lines show the inferred non-saturating [Ca^2+^] kinetics of the FHN model. (**C** and **D**): the simulated fluorescence traces (green line), membrane potentials (black line), and the applied input current (magenta line, shown in arbitrary units) to the bursting-QGIF neuron models for (**C**) *g*_*M*_ = 0.8 and (**D**) *g*_*M*_ = 0.45 *mS* / *cm*^2^. The bottom panels show the membrane potentials (posterior means; blue lines) inferred using the bursting-QGIF model for the two fluorescence traces. Parameters for simulating the data (different from Table 1): (**A**) *τ*_*Ca*_ = 600 *ms*, (**B**) *τ*_*Ca*_ = 6000 *ms*, (**C** and **D**) *I*_*app*_ = 0.74sin(0.003*t*) *μA* / *cm*^2^, *g*_*NaP*_ = 0.18, *g*_*L*_ = 0.01 *mS* / *cm*^2^, *κ*_*F*_ = 1, *K*_*d*_ = 150 *nM*, *τ*_*Ca*_ = 500 *ms*, (**C**) *g*_*M*_ = 0.8, (**D**) *g*_*M*_ = 0.45 *mS* / *cm*^2^. Parameters for inversion (different from Tables 1 and 2): (**A**) ϒ_*F*_ = 5, $\tau_{Ca}^{real}\approx 1000\;ms$, (**B**) ϒ_*F*_ = 5, $\tau_{Ca}^{real}=9000\;ms$, (**C** and **D**) $g_{M}^{0}=1\;mS/cm^{2}$ (the only free parameter, in addition to initial conditions), all other parameters were fixed at their values that we used for simulating **C** and **D**.

**I**n general the calcium/fluorescence transients may display variable kinetics. We use the parameterization of a previously reported experiment: Sasaki et al. (2008) [5] investigated the contribution of endogenous Ca^2+^ stores to calcium transients by pharmacologically depleting calcium in the endoplasmic reticulum. The authors treated the neurons with thapsigargin; an inhibitor of the Ca^2+^-ATPase family of calcium pumps. Their results showed that the thapsigargin profoundly prolongs the decay kinetics of calcium transient while its effect on the transient amplitude is insignificant. We thus reproduced such fluorescence traces (Fig 11A and 11B), first row), by using the FHN as a generative model. Based on values reported by [5], we simulated the fluorescence traces by setting *τ*_*Ca*_ = 600 *ms* for the control and *τ*_*Ca*_ = 6000 *ms* for the thapsigargin-treated neuron. We inverted the model for each generated time series. For the two inversions, we used the same priors for all parameters, as in previous sections (see Tables 1 and 2), except for two of them: Firstly, for the calcium decay time-constant we used an increased prior mean to inform the inversion about the expected effect of the pharmacological manipulation. Accordingly, we set $\tau_{Ca}^{real}$ = 1000 *ms* (consistent with calcium decay time-constant in the mammalian brain [9]) for the control and $\tau_{Ca}^{real}$ = 9000 *ms* for the thapsigargin-treated fluorescence traces, respectively. Secondly, we slightly increased the observation precision from ϒ_*F*_ = 3 to 5 in order to make the model explain the data by changing the calcium decay time-constant parameter. The results show that the inferred decay time-constants for non-saturating [Ca^2+^] kinetics (Fig 11A and 11B), second row) were reasonably well estimated as $\tau_{Ca}^{real}$ ≈ 580 *ms* and 5820 *ms*.

#### Change of voltage-gated channel conductance

In a second simulation, we tested how reliably our approach can quantify changes of the conductance of a voltage-gated channel (Fig 11C and 11D). We focused on M-type K^+^ channels, which are known to control the burstiness of action potential discharges [57,106]. Therefore, we considered an experiment based on the bursting-QGIF model in which the conductance of M-type K^+^ channels was varied (e.g. see [106]). We simulated two neurons with exactly the same parameterization (see the caption of Fig 11), except for *g*_*M*_ (set to *g*_*M*_ = 0.8 and *g*_*M*_ = 0.45 *mS* / *cm*^2^). For stimulation, we used the same sinusoidal input current for both conditions (Fig 11C and 11D), magenta lines). As expected, this input triggered a lower number of spikes per burst for higher *g*_*M*_ (2 spikes; see Fig 11C, black lines), as compared to the second condition (4 spikes, Fig 11D). Note that, as the potassium channels act and integrate effectively in (sub-) millisecond ranges, only fluorescence traces acquired with a sufficiently high temporal precision might be informative about their kinetics. This is important for inferring about the changes in *g*_*M*_. Accordingly, we subsampled the generated fluorescence traces from their original temporal precision (20 *kHz*) to a frame duration of about 0.5 *ms* (Fig 11C and 11D, green lines). Such near millisecond frame duration should be feasible with the advent of new fast imaging techniques (e.g. see [13,25]). Note that, given only these fluorescence traces, inferring about the induced changes in *g*_*M*_ is still a challenging task. This is because of the relatively similar values we used for *g*_*M*_, in combination with an indirect, highly nonlinear relationship between the activation variable of this channel and the observed fluorescence traces (see Methods). To test for a change in *g*_*M*_, we fixed all parameters at their true values (assuming that these parameters have been determined already in an initial baseline experiment), except the initial conditions and *g*_*M*_ (see the caption of Fig 11) which we kept as free parameters and for which we used the same priors as before (Table 2). This implies that the prior mean of the M-type K^+^ conductance is effectively set to $g_{M}^{0}$ = 1 *ms* / *cm*^2^ [57]. The inversions were not informed about the input current. We found that our method was able to estimate *g*_*M*_ for both conditions close to the true values as *g*_*M*_ ≈ 0.73 and 0.42 *mS* / *cm*^2^, respectively. These estimates were obtained by making a reasonably good inference about the membrane potentials (Fig 11C and 11D, blue lines), and thus the gating variable of the M-type K^+^ channels and [Ca^2+^] kinetics (not shown).

In sum, these two synthetic examples show that the new approach can infer and quantify the changes in hidden parameters of interest, given only the fluorescence traces. Particularly, the second example indicates how a focused constraint may enable the sensitive analysis of changes in biophysical parameters.

## Discussion

We have presented a novel Bayesian, biophysically informed method (called CaBBI; an abbreviation of “*calcium* imaging analysis using *biophysical* models and *Bayesian inference*”) for the analysis of calcium imaging data. Using both synthetic and *in vitro* data we have shown that CaBBI provides an accurate spike reconstruction not only under low SNR conditions but also for different fluorescence transient kinetics, such as slowly rising fluorescence transients of immature neurons. Importantly, as we used a biophysically informed method, we performed the reconstruction without an initial training phase, which is usually required for template-based methods. Using synthetic data, we have shown that the method can accurately reconstruct within-burst spikes, if the temporal resolution is high enough. As two potential applications, we have quantified the onset times of cellular activation during network events such as giant depolarizing potentials (GDPs), as well as changes in biophysically interpretable parameters due to simulated pharmacological interventions.

### Biophysical modelling

The biophysically informed model has the key advantage that the possible fluorescence trace variations are highly constrained by the equations of the generative model (see Table 3). This guards the model inference effectively against noise sources which are unlikely to be caused by noise in the modelled neuronal and calcium dynamics. In addition, CaBBI allows the incorporation of prior knowledge about the biophysically interpretable model quantities. The specification of prior distributions is a convenient compromise between fixing and freeing parameters when inverting the model. By varying the width of the prior distribution, one can effectively control how much each parameter or combinations of parameters are determined by the data or by prior knowledge. Although not shown here, formal model comparison can be used to select the best model among different prior specifications [107,108].

The biophysical modelling aspect enables CaBBI to directly infer and quantify biophysically interpretable changes caused by selective manipulations of physiological parameters using a pharmacological intervention (see Fig 11). Critically, one can test specific hypotheses by using suitable priors which are susceptible for the expected changes caused by an intervention. Using model comparison, one can proceed to test this change-sensitive model against an alternative model that does not expect this change. In this paper, we showed such an application for quantifying a particular pharmacologically-induced change: i) in the calcium decay time-constant (Fig 11A and 11B, and ii) in the conductance of M-type K^+^ channels (Fig 11C and 11D). We found that CaBBI could reasonably reliably infer about the changes in these protocols, since we appropriately fixed and/or constrained its model quantities thereby informing it about the purpose of each intervention (see the caption of Fig 11). In general, such informative constraints can augment the accuracy of corresponding inference schemes, by guarding them against over-parameterization, non-identifiability, and non-interpretability. These issues may arise from the relative complexity of the generative models (see below), and the nonlinear relationship between the observed fluorescence kinetics and the neuronal dynamics. In principle, we expect that this approach (exemplified in Fig 11) can be applied to other physiological parameters under different experimental conditions, as long as suitable model constraints are used.

To our knowledge, CaBBI is the first method which enables analyzing calcium imaging data based on biophysical models of spike and burst generations. Accordingly, in addition to proposing a pure spike reconstruction method, our aim was to establish a calcium imaging modelling framework for incorporating and inferring neuronal quantities of conductance-based neuronal networks. The main feature of CaBBI is that, using the same parameterization, it can model fluorescence transients with rather different kinetics. Such variability can, in principle, render the spike reconstruction difficult for methods which are based on fixed or prototypical templates, e.g. [7,9,12–14,17,109]. In contrast, CaBBI, similar to some previously proposed methods [19,25,71], does not rely on a fixed template but can adapt itself to the transient kinetics of each individual neuron in order to precisely reconstruct spikes. We showed this for fluorescence traces containing transients with significantly variable kinetics, i.e. with rather inhomogeneous rise and decay kinetics (e.g. see Fig 10). The reason for this adaptation ability is that CaBBI is informed by the generative biophysical model about the possible kinetics and infers the exact kinetics from the data. Therefore, CaBBI does not require an initial training phase neither for constraining the prior distributions [25] nor for setting the optimal method parameters manually.

One potential limitation is that CaBBI, as compared to alternative methods, requires rather long computer run times due to the Bayesian inference and the implementation in Matlab. For example, the reconstruction of the spikes of a single fluorescence trace with 6,000 frames using default parameters and the QGIF model requires less than an hour on a standard desktop computer. To compute all inversions reported in this paper in an acceptable time, we made use of compute servers. A second potential limitation is that due to the relative complexity of the generative models we had to fix many of the parameters at some suitable values reported by previous experimental and modelling studies (see Table 1). Without these constraints or using proper prior distributions (see Table 2), CaBBI may be too unconstrained. In this paper, we demonstrated that such suitable constraints exist for the generative models and data we have used. Finally, the current generative models of CaBBI are not able to reconstruct the spike counts from the burst-evoked transients which have similar shape as single-spike-evoked transients, e.g. due to a low sampling rate. For adult neurons, usually, such transients differ mainly in their amplitudes depending on the spike counts of their underlying events. Although only a few methods (like the fast filter [71]) are, at least in principle, able to decode the bursts’ spike counts from such variability (e.g. in the amplitudes), our current generative models treat all such transients as having been evoked by single spikes (e.g. see Fig 9). This is because of the biophysical essence of our neuron models, which relate each resolved transient to a spike, and compensate for such variability in the rise kinetics through a proper regulation of the neuronal dynamics.

### FHN and QGIF models

For CaBBI to be applied, one requires a biophysically informed model based on continuous over time, differentiable dynamics. In the literature, a wide range of both spiking and bursting models was reported [21], including Hodgkin-Huxley-type models [38], Morris-Lecar model [110], and Hindmarsh-Rose model [36]. Another criterion for CaBBI to work properly is that the generative model should have a rather low number of variables and parameters as the fluorescence trace is not too informative about the underlying neuronal variables due to the temporal smearing of the calcium responses. As a representative of such continuous models we have selected the widely-used FHN model (2D), which is able to produce single spikes [40–42]. Alternatives would have been analogous 2D models such as the Morris-Lecar model [110] or reduced versions of the Hodgkin-Huxley model [111]; see also [49]. We also used integrate-and-fire (I&F) models. To make them continuous and avoid the discontinuous reset conditions of I&F models, e.g. [33,112,113], we described two new models called QGIF (1D) and bursting-QGIF (2D), see Table 3. These two models adopt a minimum number of required variables for producing the single (QGIF) and burst (bursting-QGIF) spiking patterns. In addition, the QGIF model can be readily extended to describe different types of neurons with their specific active ionic currents [114], e.g. we showed this by creating the bursting-QGIF model for hippocampal pyramidal neurons.

With respect to the reconstruction efficiency we found that the FHN model is better suited for more accurate single spike and event (like GDP occurrence) detection than the QGIF model. Under a low SNR, the inferred membrane potentials of the QGIF model were usually relatively noisy and the inferred spikes sometimes did not cross the detection threshold (see Methods) of zero, and were thus counted as missed events (e.g. see Table 4); this indicates that to increase the detection accuracy of the QGIF model one may need to use a lower detection threshold, like -10 *mV*. The greater robustness of the FHN model is possibly due to its recovery variable (Eq 2) whose negative feedback on membrane potential constrains the membrane potential kinetics.

For fluorescence transients with slow rise kinetics observed in our experimental data, we found that the QGIF model can reconstruct the onset times of GDP events more accurately than the FHN model (see Fig 8, and text). The precise GDP onset time reconstruction of the QGIF model was preserved even for low SNR traces. For the QGIF model, the optimization process converges usually quicker than the FHN model. Therefore, in terms of time consumption the QGIF model can be more reasonable to use for data of, e.g., a population of neurons imaged by regular experimental setups which usually acquire data at rather high SNR levels.

### Further improvements and extensions

An advantage of CaBBI is that one can readily modify the generative models or replace them with other models. For instance, using CaBBI for the reconstruction of spikes from slowly rising fluorescence transients acquired at near-millisecond temporal resolution may further require a modification of the generative models. To do this, one can incorporate, e.g., an extended model of calcium dynamics (Eq 11) which prolongs the Ca^2+^ influx so that the mechanism of delay between the spike occurrence and the fluorescence transient peak is explicitly captured. For data providing such temporal resolution, one can also model the experimentally observed double-exponential decay kinetics of calcium transients including the typically observed rise time [13,115]. In general, this observation reflects the contribution of two [Ca^2+^] decay mechanisms with different time-constants, which can be modeled in Eq 11, similarly to [115]. This generalization should provide a better fit to data showing such kinetics, and thus enhance the reconstruction precision.

Large-scale calcium imaging from populations of individual neurons aims to provide a better understanding of neuronal circuit dynamics [1,7,11]. CaBBI provides a new model-based calcium imaging framework, and as an outlook, may be extended to analyze the data of imaged populations. More specifically, the presented approach is a first step towards incorporating the networks of biophysical spiking neuron models (e.g. see [116,117]), together with their ubiquitous mechanisms such as synaptic plasticity (e.g. see [117–119]). We expect that such a network extension enables studying the neuronal dynamics and biophysical parameters of complex neuronal circuits measured indirectly by calcium imaging.

## Supporting Information

S1 Appendix Model specifications and parameters. (DOCX)

S2 Appendix Polynomial filter. (DOCX)

S1 Fig Additional illustrative results of the proposed approach for *in vitro* fluorescence traces with slowly rising transients. The same format is used as in Fig 9. This figure shows the inferred membrane potentials (posterior means) when using the FHN and QGIF models for two representative *in vitro* fluorescence traces with slowly rising transients evoked by GDP-mediated single spikes, under low SNR.
(TIF)
