# Correction: Inferring Neuronal Dynamics from Calcium Imaging Data Using Biophysical Models and Bayesian Inference

**DOI:** 10.1371/journal.pcbi.1004835

**Published:** 2016-03-11

**Authors:** The PLOS Computational Biology Staff

Table 3 has been typeset incorrectly. The publisher apologizes for this error.

Please view the correct version of Table 3 here:

**Table 3:** Generative models.

		Spiking models		Calcium channel		[Ca^2+^] kinetics		Ca^2+^ -fluorescence mapping		Generative model’s name
Eqn. 1	**{**	**QGIF** (Eqs. 3–6)	+	Eqs. 9–10	+	Eqn. 11	+	Eqn. 12	**}** =	**QGIF**
Eqn. 1	**{**	**Bursting-QGIF** (Eqs. 4–7)	+	Eqs. 9–10	+	Eqn. 11	+	Eqn. 12	**}** =	**Bursting-QGIF**
Eqn. 1	**{**	**FHN** (Eqn. 2)	+	Eqs. 9–10	+	Eqn. 11	+	Eqn. 12	**}** =	**FHN**
