# Supplementary material for: Inferring Neuronal Dynamics from Calcium Imaging Data Using Biophysical Models and Bayesian Inference
Source: PLoS Comput Biol. 2016 Feb 19;12(2):e1004736. doi: 10.1371/journal.pcbi.1004736 (PMC4760968; doi:10.1371/journal.pcbi.1004736)
Supplement: S1 Appendix — (DOCX) [file pcbi.1004736.s001.docx]

# **S1 Appendix**

Rahmati, Vahid; Kirmse, Knut; Marković, Dimitrije; Holthoff, Knut; Kiebel, Stefan J.

# **Model specifications and parameters.**

In this appendix, we provide a collection of useful detail regarding some features and characteristics of the models adopted by the evolution equations, as follows.

(i) **Spiking models**. For the FHN model, variables $V$ and $W$ have arbitrary physical units. For the QGIF model, the parameters $V_{peak}$ and $\bar{I}_{rep}$ can be adjusted in order to control the peak of spike and its repolarization size, respectively. In our parameterization, by setting $V_{peak}$= 30 $mV$, the model generates spikes with the peak around 30 mV, as reported for hippocampal pyramidal neurons (see [58,29] in the main text). For the bursting-QGIF model, specifying appropriate values to, for example, $\bar{I}_{rep}$, $V_{peak}$, $g_{M}$ and $g_{NaP}$ makes the model amenable to complex firing patterns, which resemble a variety of physiological behaviors, e.g. see [57,106] in the main text.

(ii) **Model of calcium dynamics**. For the HVA calcium channels, we set $V_{1/2}$= -25 $mV$ (see [64] in the main text) for simulating the data and $V_{1/2}$= -45 $mV$ (see [67] in the main text) for the inversions, thereby approximating the Cav 1.2 and Cav 1.3 forms of L-type calcium channels (see [65] in the main text). Besides, by decreasing $V_{1/2}$ the inversions were facilitated due to requiring less state-noise for spike reconstruction. In addition, note that in our formulations calcium traces account for the deviation of $[\text{Ca}^{2+}]$ from $[\text{Ca}^{2+}]_{\text{base}}$ (see Eqn. 11). Accordingly, in the simulations and inversions we set $[\text{Ca}^{2+}]_{\text{base}}$= 0 and $\mu_{[\text{Ca}^{2+}]_{\text{base}}}$= 0, respectively. The inference about $[\text{Ca}^{2+}]_{\text{base}}$ should, however, reflect the changes in the actual rest value of $[\text{Ca}^{2+}]$, which is reported to be between 50 and 100 $nM$ (see [77] in the main text). Consistent with this, we have already added the inferred non-saturating $[\text{Ca}^{2+}]$ kinetics (shown in Results) by 50 $nM$; our choice for the value of rest $[\text{Ca}^{2+}]$. Here, as an arbitrary physical unit, we considered $[\text{Ca}^{2+}]$ in units of $[nM]$, though as we showed in Results section, this choice can put the simulated and inferred $[\text{Ca}^{2+}]$ kinetics in biologically plausible ranges.
