# Supplementary material for: Inferring Neuronal Dynamics from Calcium Imaging Data Using Biophysical Models and Bayesian Inference
Source: PLoS Comput Biol. 2016 Feb 19;12(2):e1004736. doi: 10.1371/journal.pcbi.1004736 (PMC4760968; doi:10.1371/journal.pcbi.1004736)
Supplement: S2 Appendix — (DOCX) [file pcbi.1004736.s002.docx]

# **S2 Appendix**

Rahmati, Vahid; Kirmse, Knut; Marković, Dimitrije; Holthoff, Knut; Kiebel, Stefan J.

# **Polynomial filter.**

To model the slowly varying drifts in the fluorescence traces we used a fourth degree polynomial basis function:

| $Pl\left(F_{t}\right)=\varpi_{0}+\varpi_{1}F_{t}+\varpi_{2}F_{t}^{2}+\varpi_{3}F_{t}^{3}+\varpi_{4}F_{t}^{4}$ |
| --- |

where $\varpi_{i}$ ($i$= 0, …, 4) are constant coefficients, and $F_{t}$ is the given fluorescence trace. This function is fitted (using a least-squares method) to the given fluorescence trace so that the values of coefficients can be estimated. Afterwards, the detrended fluorescence trace is acquired by subtracting the fitted function from the given fluorescence trace.
