# Supplementary figures and images for: Inferring Neuronal Dynamics from Calcium Imaging Data Using Biophysical Models and Bayesian Inference
Source: PLoS Comput Biol. 2016 Feb 19;12(2):e1004736. doi: 10.1371/journal.pcbi.1004736 (PMC4760968; doi:10.1371/journal.pcbi.1004736)

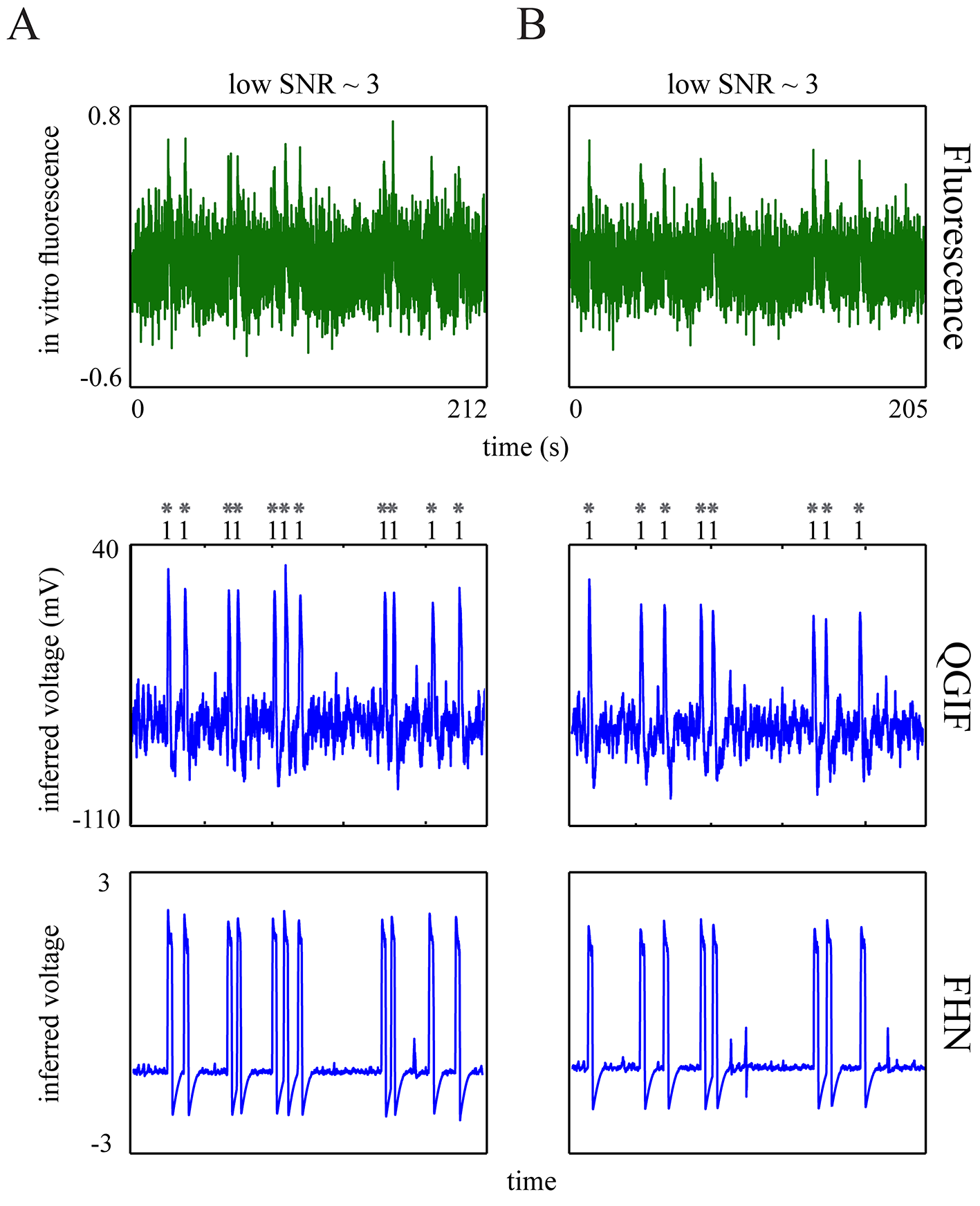

Supplement: S1 Fig — The same format is used as in Fig 9. This figure shows the inferred membrane potentials (posterior means) when using the FHN and QGIF models for two representative in vitro fluorescence traces with slowly rising transients evoked by GDP-mediated single spikes, under low SNR. (TIF) [file pcbi.1004736.s003.tif]
